# Immunomodulatory proteins from hookworms reduce cardiac inflammation and modulate regulatory responses in a mouse model of chronic *Trypanosoma cruzi* infection

**DOI:** 10.3389/fpara.2023.1244604

**Published:** 2023-10-12

**Authors:** Kathryn M. Jones, Bin Zhan, Keenan J. Ernste, Maria Jose Villar, Nalini Bisht, Duc Nguyen, Li-Yen Chang, Cristina Poveda, Gonteria J. Robinson, Akshar J. Trivedi, Colby J. Hofferek, William K. Decker, Vanaja Konduri

**Affiliations:** 1 National School of Tropical Medicine, Department of Pediatrics, Baylor College of Medicine, Houston, TX, United States; 2 Texas Children’s Hospital Center for Vaccine Development, Houston, TX, United States; 3 Department of Pathology & Immunology, Baylor College of Medicine, Houston, TX, United States; 4 Center for Comparative Medicine, Baylor College of Medicine, Houston, TX, United States; 5 Department of Medical Microbiology, Universiti Malaya, Kuala Lumpur, Malaysia; 6 Molecular & Human Genetics Department, Baylor College of Medicine, Houston, TX, United States; 7 Dan L. Duncan Cancer, Baylor College of Medicine, Houston, TX, United States; 8 Center for Cell and Gene Therapy, Baylor College of Medicine, Houston, TX, United States

**Keywords:** hookworm, anti-inflammatory, *Trypanosoma cruzi*, myocarditis, immunomodulatory

## Abstract

**Introduction:**

Hookworms are parasitic helminths that secrete a variety of proteins that induce anti-inflammatory immune responses, stimulating increased CD4 + Foxp3+ regulatory T cells and IL-10 production. Hookworm-derived recombinant proteins AIP-1 and AIP-2 have been shown to reduce inflammation in mouse models of inflammatory bowel disease and inflammatory airway disease by inducing CD4+Foxp3+ cells and IL-10 production. In contrast, chronic infection with the protozoal parasite *Trypanosoma cruzi*, the causative agent of Chagas disease, leads to chronic inflammation in tissues. Persistence of the parasites in tissues drives chronic low-grade inflammation, with increased infiltration of inflammatory cells into the heart, accompanied by increased production of inflammatory cytokines. There are no current antiparasitic drugs that effectively reduce or prevent chronic myocarditis caused by the onset of Chagas disease, thus new therapies are urgently needed. Therefore, the impact of AIP-1 and AIP-2 on myocarditis was investigated in a mouse model of chronic *T. cruzi* infection.

**Methods:**

Female BALB/c mice infected with bioluminescent *T. cruzi* H1 strain trypomastigotes for 70 days were treated once daily for 7 days with 1mg/kg AIP-1 or AIP-2 protein by intraperitoneal injection. Control mice were left untreated or treated once daily for 14 days with 25mg/kg aspirin in drinking water. At 84 days of infection, splenocytes, cardiac tissue and serum were collected for evaluation.

**Results:**

Treatment with both AIP-1 and AIP-2 proteins significantly reduced cardiac cellular infiltration, and reduced cardiac levels of IFNγ, IL-6 and IL-2. AIP-2 treatment reduced cardiac expression of COX-2. Further, while incubation with AIP-1 and AIP-2 proteins did not induce a significant upregulation of an immunoregulatory phenotype in dendritic cells (DC), there was a modest upregulation of CD11c +CD11b+MHCII+SIRPα+ expression, suggesting a regulatory phenotype. *Ex-vivo* stimulation of splenocytes from the treatment groups with AIP-1 loaded DC induced reduced levels of cytotoxic and pro-inflammatory T cells, stimulation with AIP-2 loaded DC specifically induced enhanced levels of CD4+CD25+Foxp3+ regulatory T cells among treatment groups.

**Discussion:**

All *in vivo* and *in vitro* results demonstrate that hookworm-derived AIP-1 and AIP-2 proteins reduce *T. cruzi* induced cardiac inflammation, possibly through multiple anti-inflammatory mechanisms.

## Introduction

Over long-term co-evolution in the host, helminths have developed sophisticated mechanisms to evade host immunity and establish chronic infections through secreting proteins with immunomodulatory functions (van Riet et al., 2007; McSorley et al., 2013). These immunomodulatory proteins manipulate host immune responses by inducing T_H_2-bias as well as inducing production of IL-10 and TGFβ cytokines that promote regulatory T cell responses (Nair and Herbert, 2016). This regulatory response inhibits host inflammatory responses that would promote helminth expulsion, thereby ensuring long-term survival of the parasites and chronic infection (van Riet et al., 2007). While this is an evolutionary advantage for survival of the parasite, it provides an added benefit of protection for the host against other inflammatory diseases (McSorley et al., 2013). The immunoregulatory prowess of helminth infections has been exploited to develop novel therapeutics for preclinical models of allergic and autoimmune inflammatory diseases, ultimately providing significant relief from severe clinical disease (Elliott et al., 2007; Hübner et al., 2012; Li et al., 2017). Hookworms are blood-feeding intestinal nematode parasites that secrete an abundance of proteins at attachment sites on the intestinal mucosa, the interface between the parasite and the host, to facilitate feeding and suppress host immune responses as a survival strategy. Controlled hookworm infections have been used in clinical trials for treatment of inflammatory intestinal conditions, including inflammatory bowel disease and celiac disease (Croese et al., 2006; Croese et al., 2015). Hookworm secreted proteins have also been effective at inducing regulatory responses and suppressing allergic inflammatory and autoimmune conditions in animal models (Ruyssers et al., 2009; Ferreira et al., 2013). Among hookworm secreted proteins, we previously identified two tissue inhibitor of metalloprotease (TIMP)-like proteins, designated Ac-TIMP-1 and Ac-TIMP-2, as major proteins secreted by the adult dog hookworm *Ancylostoma caninum* (Zhan et al., 2002; Zhan et al., 2008). *In vitro*, Ac-TIMPs induced a regulatory phenotype in bone marrow-derived dendritic cells (BMDC) with increased production of IL-10 and TGFβ. Further, naïve CD4+ and CD8+ T cells incubated with Ac-TIMP-1 treated BMDC induced differentiation of both CD4+ and CD8+CD25+Foxp3+ T cells that expressed IL-10, and functionally suppressed proliferation of both naïve and activated CD4+ cells *in vitro* (Cuellar et al., 2009). Hookworm-derived TIMP-1 was later redesignated as anti-inflammatory protein-1 (AIP-1), and the homologous protein from the human hookworm *Necator americanus* (Na-AIP-1) reduced acute inflammation in the colon in a mouse model of trinitrobenzoylsulfonic acid-induced colitis in a CD11c+ cell-dependent manner (Ferreira et al., 2017; Buitrago et al., 2021). Intraperitoneal (IP) administration of Na-AIP-1 increased the production of IL-10 and TGFβ, and reduced TNFα, IL-13 and IL-17A production while reducing COX-2 expression (Ferreira et al., 2017; Buitrago et al., 2021). Similarly, Ac-TIMP-2, redesignated as AIP-2, administered by IP injection induced mesenteric CD103+ regulatory DC and Foxp3+ regulatory T cells which reduced airway inflammation in a mouse model of allergic airway disease (asthma) (Navarro et al., 2016). Together, these data confirm that hookworm-secreted AIP-1 and AIP-2 perform immunomodulatory functions to exert anti-inflammatory effects in the host.

In contrast to helminth infection, chronic infection with the protozoal parasite *Trypanosoma cruzi* causes an inflammatory cardiomyopathy which results in scarring and gross changes in cardiac morphology, clinically known as chronic Chagasic cardiomyopathy (CCC) (Rossi et al., 2003; Alvarez et al., 2014). Invasion of parasites into cardiomyocytes causes cellular hypertrophy and recruitment of inflammatory cells into tissues, including macrophages, neutrophils, CD4+ and CD8+ T cells (Higuchi Mde et al., 1993; Higuchi et al., 1997). Pro-inflammatory cytokines, including IFNγ, TNFα and IL-6, are elevated in cardiac tissue and in the serum of patients with CCC and correlate with disease severity (Reis et al., 1997; Laucella et al., 2004; Guedes et al., 2012; Magalhães et al., 2013; Sousa et al., 2014; Sousa et al., 2017). Additionally, a recent study showed that CD4+CD25^high^Foxp3+ regulatory T cells (Tregs) were significantly reduced, while cytotoxic CD4+ cells were significantly increased in CCC patients compared to healthy controls (Almeida et al., 2022). Overall, appropriate host immune responses are critical for controlling both the parasite burden and the resulting inflammatory response that leads to tissue damage and dysfunction. Initially, a T_H_1-driven immune response with high levels of IFNγ and TNFα is essential for activating parasite killing mechanisms (Wirth et al., 1985; Silva et al., 1992; Vespa et al., 1994; Aliberti et al., 1996; Camargo et al., 1997). Further, parasite-specific effector CD8+ T cells and macrophages (Mφ) infiltrate the heart and eliminate extracellular parasites as well as cardiac cells harboring intracellular parasites (Tarleton, 1990; Abrahamsohn and Coffman, 1996; Tzelepis et al., 2006; Tarleton, 2007). Classically activated M1 Mφs are efficient at phagocytosis and killing pathogens at sites of infection, by upregulation of inducible nitric oxide synthase (iNOS) and subsequently nitric oxide (NO) production in response to inflammatory cytokines (Sheel and Engwerda, 2012; Ginhoux and Guilliams, 2016). Additionally, inflammatory processes are mediated in part by cyclooxygenases (COX) enzymatic activity (Thwe and Amiel, 2018). Two isoforms are well characterized and designated COX-1 and COX-2, with COX-1 being constitutively active while COX-2 is induced at sites of inflammation (Dubois et al., 1998). It has been previously shown that acute *T. cruzi* infection induces inflammatory markers in cardiac tissue, including endothelin-1 and COX-2 expression (Corral et al., 2013). Importantly, it has been shown that pharmacologic inhibition of COX-1 and COX-2 enzymatic activity significantly inhibited invasion of both cardiac cells and macrophages by *T. cruzi in vitro*, concomitantly enhancing the production of NO (Malvezi et al., 2014a; Malvezi et al., 2014b). Thus, novel therapeutics that target key inflammatory pathways induced by *T. cruzi* could produce desirable therapeutic outcomes. After sufficient control of *T. cruzi* burden is achieved, the regulatory immune response, with increased production of IL-10, IL-17 and Tregs is necessary to control tissue inflammation and prevents further damage (Mariano et al., 2008; Santos et al., 2020). These data suggest that in chronic *T. cruzi* infections, preservation of cardiac function is at least partially dependent on effective regulation of inflammatory immune responses that induce tissue damage. Importantly, a key proof-of-concept study in mice experimentally co-infected with the helminth parasites *Schistosoma mansoni* and *T. cruzi* showed that cardiac inflammation was significantly decreased in co-infected mice compared to *T. cruzi*-only infected mice (Rodrigues et al., 2017). Further, co-infected mice had significantly higher expression of IL-10 and lower IFNγ in the heart compared to *T. cruzi*-only infected mice (Rodrigues et al., 2017). Together, these data provide strong evidence that helminth derived proteins have immunomodulatory effects that can reduce inflammatory responses in both infectious and non-infectious inflammatory diseases.

In the current proof-of-principle study, we investigated the role of hookworm-derived proteins AIP-1 and AIP-2 in reducing cardiac cellular infiltrate, production of cardiac pro- and anti-inflammatory cytokines, and expression of inflammatory genes in a mouse model of chronic *T. cruzi* infection. We also studied the role of these proteins in modulating the phenotype of antigen-presenting DC and the impact of those DC in modulating downstream T cell responses.

## Materials and methods

### Ethics statement

Animal experiments were performed in full compliance with the Public Health Service Policy and the National Institutes of Health Guide for the Care and Use of Laboratory Animals, 8th edition, under a protocol approved by Baylor College of Medicine’s Institutional Animal Care and Use Committee, assurance number D16-00475 (Council, N. R, 2011).

### Mice and parasites

Female BALB/c mice (BALB/cAnNTac) and male ICR-SCID (ICRSC-M) were obtained at 5-6 weeks of age from Taconic (Taconic Biosciences, Inc) and allowed to acclimate for one week prior to any manipulation. Mice were housed in groups of 4 in small microisolator caging, with *ad libitum* food and water and a 12 hr light/dark cycle. Male ICR-SCID mice were infected with 5000 blood form bioluminescent *T. cruzi* H1 trypomastigotes transfected with the pTRIX2-RE9h plasmid containing PpyRE9h, the thermostable, red-shifted firefly luciferase gene (Ruiz-Sanchez et al., 2005; Branchini et al., 2010; Lewis et al., 2015), by IP injection. Blood form trypomastigotes were collected at approximately 28 days of infection to generate sufficient virulent parasites for experimental infections.

### Expression and purification of recombinant protein

DNA encoding Ac-AIP-1 (AIP-1) (GenBank accession number AF372651) with a hexa-histidine tag added at the C-terminus was amplified from total cDNA of adult *A. caninum* worms and cloned into the *Pichia pastoris* expression vector pPICZαA (Invitrogen) using XhoI and XbaI restriction sites. DNA encoding Ac-AIP-2 (AIP-2) (GenBank accession numbers: EU523698) was codon optimized based on yeast codon usage preference and synthesized by GenScript (NJ, USA) before being cloned into pPICZαA without any tag expressed. The correct open reading frame (ORF) was confirmed by sequencing using the vector flanking primers α-factor and 3’AOX1 genes. Both recombinant AIP proteins were expressed in *P. pastoris* X-33 under induction of 0.5% methanol for 72 hours. Secreted AIP-1 was purified from culture supernatant by immobilized metal affinity chromatography (IMAC) and AIP-2 was purified by HiTrap QXL anion exchange (ThermoFisher). The purified proteins were buffered to PBS, pH 7.4 by dialysis and stored at −80°C.

### Preparation of parasite lysate

Soluble lysate from bioluminescent *T. cruzi* parasites was prepared as previously described (Villanueva-Lizama et al., 2020). Briefly, bioluminescent H1 *T. cruzi* trypomastigotes were harvested from tissue culture and washed, followed by four freeze-thaw cycles. The solution was then sonicated three times for 15 seconds and cooled on ice for 10 seconds. Parasite lysate was collected from the supernatant after centrifugation at 14,000 x g for 30 minutes. The lysate concentration was determined by Pierce BCA Protein Assay Kit (ThermoFisher).

### Preparation of bone marrow-derived dendritic cells

Bone marrow cells were flushed from BALB/c mouse tibia and femur and cultured in AIM-V containing 10% fetal bovine serum (FBS) and 1% penicillin-streptomycin-amphotericin antibiotic-antimycotic (anti-anti) (Invitrogen) supplemented with 50ng/mL murine granulocyte-macrophage colony-stimulating factor (mGM-CSF) and 10ng/mL murine interleukin-4 (mIL-4) (R&D Systems) for 3 days. Cells were cultured in a humidified chamber at 37°C with 5% atmospheric CO2. The culture medium was removed and replenished with an equal volume of fresh medium supplemented with cytokines on day 3. On day 5, the cells were replenished with fresh AIM-V containing 10% mouse serum and 1% anti-anti and supplemented with additional mGM-CSF to 50ng/mL and mIL-4 to 10ng/mL. Immature DC were harvested on day 6, counted, and incubated with 50μg/mL hookworm derived AIP-1 (herein referred as AIP-1 DC) or AIP-2 (AIP-2 DC) proteins or 10ng/mL *T. cruzi* parasite derived lysate (*T. cruzi* DC) for three hours in AIM-V media supplemented with 5% mouse serum as described previously (Konduri et al., 2016; Konduri et al., 2017; Liang et al., 2017). After 3 hours of antigenic incubation, DC were gently washed with PBS, allowed to mature for 48 hours in AIM-V supplemented with 10% mouse serum and a pro-inflammatory cytokine cocktail of 50ng/mL GM-CSF, 10ng/mL IL-4, 10ng/mL IL-1β (R&D Systems), 10ng/mL tumor necrosis factor alpha (TNFα) (R&D Systems), 15ng/mL IL-6 (R&D Systems), and 1μg/mL prostaglandin E2 (PGE2) (Sigma-Aldrich). Control DC were not loaded with *T. cruzi* lysate, AIP-1 or AIP-2 proteins but were matured with pro-inflammatory cytokine cocktail.

### Aspirin treatment

Following the protocol by Molina-Berrios et al (Molina-Berrıos et al., 2013), all mice were weighed prior to initiation of treatment and the average weight was calculated at approximately 25g (0.025kg). Mice were observed to consume approximately 4mL of water per day *ad libitum.* Based on these observations, aspirin (acetylsalicylic acid, Sigma-Aldrich) was dissolved to a final concentration of 160μg/mL in deionized water and provided *ad libitum* to mice in drinking water to achieve an approximate dose of 25mg/kg.

### Study design

**FIGURE 1:**
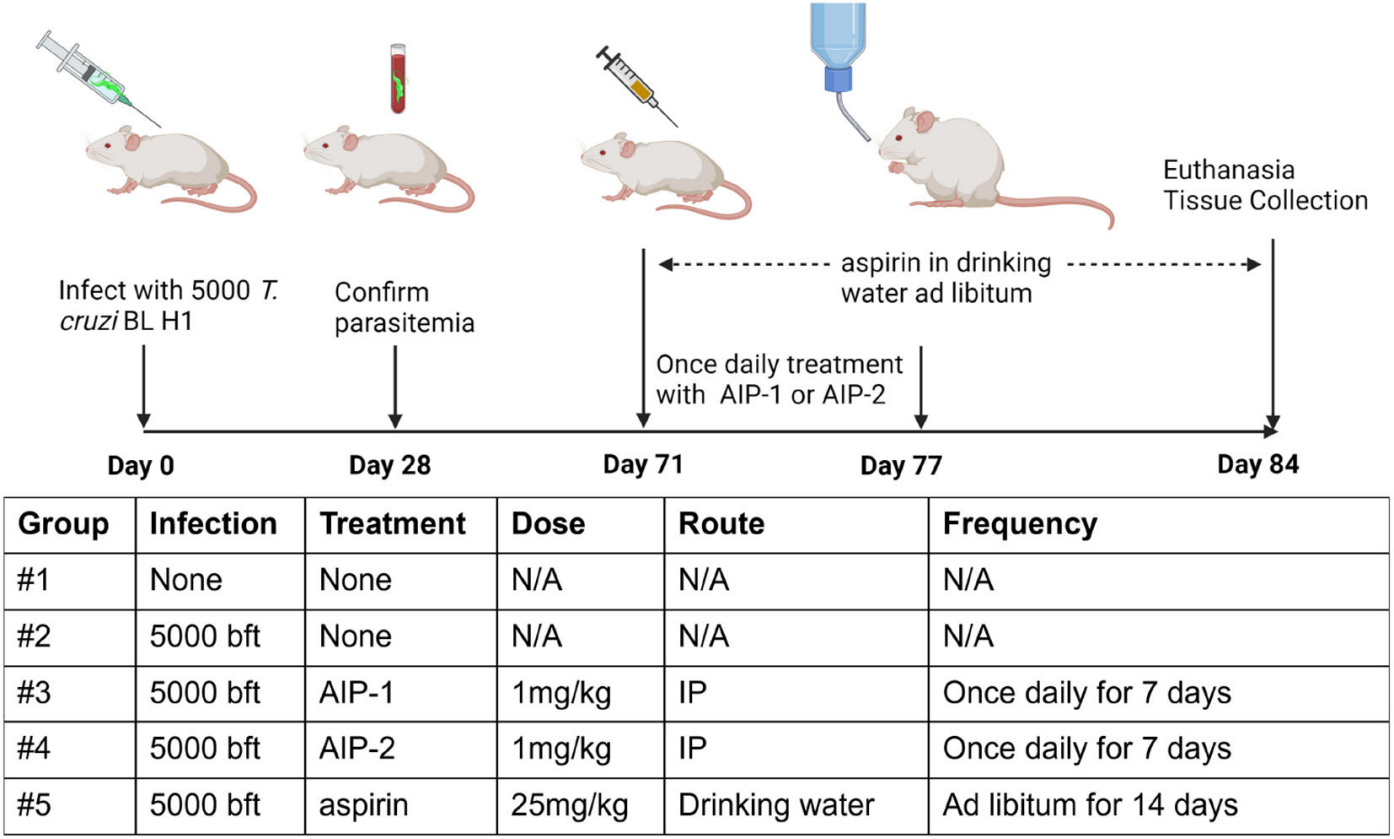
Study timeline and treatments. Bft stands for Blood form trypomastigotes. N/A, Not Applicable. Image created with Biorender.

BALB/c mice were infected with 5000 bioluminescent blood form *T. cruzi* H1 trypomastigotes (bft) by IP injection. Naïve age-matched mice were left uninfected as controls. Blood was collected by tail vein microsampling from all mice at approximately 28 days post infection (DPI) to confirm parasitemia by quantitative PCR. After approximately 70 days post infection, mice were weighed and randomly assigned to treatment groups of n=10 mice each. Mice were treated with 1mg/kg recombinant AIP-1 or AIP-2 protein by IP injection once daily for 7 days, 25mg/kg aspirin in drinking water *ad libitum* for 14 days or left untreated (Figure 1). All mice were humanely euthanized at 84 days post infection, and whole blood, hearts, and spleens were collected for *in vitro* analyses.

Samples collected at the endpoint were evaluated for parasitemia and parasite burden as well as immunological assays including gene expression by RT-PCR, Luminex cytokine analysis, histopathology, ELISA, and co-culture of splenocytes with dendritic cells. Approximately 5μL of blood was added to 100μL of nuclease-free water and stored in −20°C. Cardiac tissues were cut in half and one-half was utilized for histopathologic analysis while the other half was trimmed into three sections and stored frozen at −80°C for further processing.

### ELISA for serum antibody levels

Whole blood was collected into serum separator tubes and allowed to clot at room temperature (RT) for at least 30 minutes. Tubes were centrifuged at 10,000xg for 5 minutes at RT to separate serum, after which serum was transferred to a cryovial and frozen at −80°C until use in ELISA assays. To measure the mouse antibody response after receiving treatment of AIP-1 and AIP-2 (Navarro et al., 2016), 96-well Nunc-Immuno Maxisorp plates (ThermoFisher) were coated with 100μL of recombinant AIP-1 or AIP-2 at a concentration of 5.0μg/mL in coating buffer (KPL) overnight at 4°C. The coated plates were blocked with 1.0% BSA in PBST (PBS +0.05% Tween-20), then incubated with 2-fold serially diluted serum samples, starting at 1:200 dilution, in 0.1% BSA in PBST for 2 hours at room temperature. Horseradish peroxidase (HRP)-conjugated goat anti-mouse IgG (1:6,000 in PBST) was used as secondary antibody. Sure Blue TMB (KPL) was added as the substrate. The reaction was stopped by adding 100μL 1M HCl, and the absorbance was measured at 450nm using a spectrophotometer (BioTek).

### Parasitemia and parasite burden

DNA was extracted from blood and sections of frozen cardiac tissue using the PDQeX Nucleic Acid Extractor and PreGem Universal kits (MicroGem International) according to the manufacturer’s protocol. Blood and tissue levels of *T. cruzi* were quantified using Taqman primers and probes for the *T. cruzi* satellite DNA and mouse GAPDH (Supplemental Table 1) as previously described (Piron et al., 2007; Villanueva-Lizama et al., 2020). Samples were amplified using a QuantStudio 3 real time thermocycler (Applied Biosystems). The observed CT mean for each sample was normalized to calculate the parasite per mL blood and parasite burden per mg tissue using the equation derived from the standard curve.

### RNA extraction and RT-PCR

Total RNA was isolated from frozen cardiac tissue by homogenization with gentleMACS M Tubes (Miltenyi Biotech) followed by RNA isolation using the RNeasy Mini Kit (Qiagen), following the manufacturers’ protocols. Total RNA was quantified with the NanoDrop 2000C Spectrophotometer (Thermo Fisher) and was diluted to 100ng/μL for RT-PCR. Diluted RNA was reverse transcribed using High-Capacity cDNA Reverse Transcription Kit (Thermo Fisher) and T100 Thermocycler (Bio Rad). The cDNA was then diluted 10-fold and gene expression evaluated using the QuantStudio 3 (Applied Biosystems) for qPCR. Genes of interest were evaluated using Taqman (Life Technologies) primers and probes as shown in Supplemental Table 2. Gene expression levels were measured using the comparative Ct method. The difference between the observed Ct mean of each gene and the GAPDH (ΔCt Mean) was calculated followed by the difference between the averaged Ct mean of the naïve group as it is used as a blank (ΔΔCt Mean). Copy number determination and gene expression levels were then calculated by 2^-(ΔΔCt Mean).

### Cardiac lysate and Luminex

To obtain total proteins, cardiac lysate was isolated from frozen sections of cardiac tissue by lysis in RIPA Lysis and Extraction Buffer (Thermo Fisher) followed by homogenization using gentleMACS M Tubes (Miltenyi Biotech). The collected lysate was diluted 1:10 to prepare sample with a final concentration of 250μg for quantification using a Pierce^™^ BCA Protein Assay Kit (Thermo Fisher). Both cardiac lysate and sera were evaluated for IFNγ, TNFα, IL-10, IL-4, IL-17, IL-13 using MILLIPLEX MAP Mouse Cytokine/Chemokine Magnetic Bead Panel (Millipore Sigma). Cytokine levels were determined by the observed concentration in each sample using the Minimal Detectable Concentrations (MinDC) indicated in the manufacturer’s instructions. If the observed concentration was lower than the MinDC + 2SD, the value was set as the MinDC.

### Histopathology

Formalin-fixed paraffin embedded tissues were sectioned for histopathology analysis. Sections were stained with Hematoxylin & Eosin (H&E) and Picrosirius red to analyze for inflammation and fibrosis, respectively. Three representative images of the left ventricles were taken of each slide at 10x magnification using the AmScope 40X-2000X Professional Biological Microscope (AmScope). Images were analyzed for inflammation and fibrosis using the Fiji Image J software (National Institutes of Health). White background was removed from the image total pixels (18095808) to determine the tissue fraction and then the respective staining colors were selected. Inflammation was determined by the selection of purple nuclei whereas fibrosis was selected as blue regions within the cardiac tissue.

### Splenocyte preparation and stimulation

Spleens were harvested from the mice (n=10) from each of the five treatment groups (uninfected, infected untreated and infected treated with AIP-1, AIP-2, aspirin). Harvested spleens were further combined into 3 biological replicates (3 spleens for replicate 1, 3 spleens for replicate 2 and 4 spleens for replicate 3) per group. After thorough homogenization, combined splenocytes were filtered, counted using hemocytometer by diluting the cells in trypan blue and cultured together with mature BMDC (Control DC, AIP-1 DC, AIP-2 DC and *T. cruzi DC*) at a ratio of 10:1 (Splenocytes: DC) in 24-well flat-bottomed tissue culture plates with RPMI-1640 supplemented with 10% mouse serum. 250,000 DC were cultured with 2.5 million splenocytes per well. Culture supernatants were collected after 48 hours of co-culture for serum cytokine analysis by Luminex. After five days of co-culture, the cells were supplemented with 50Units/mL murine IL-2 every alternate day followed by stimulation with the respective mature DC on day 9. While stimulation with *T. cruzi-loaded* DC served as antigenic re-challenge, stimulation with AIP-1 and AIP-2 loaded DC served as a treatment boost. IL-2 was added again on day 10 and every alternate day until day 13.

### Flow cytometry analysis

Samples were analyzed on either an LSR II or LSR Fortessa flow cytometer with a high throughput sampler attachment (BD Biosciences). Panels of the fluorochrome-conjugated antibodies used for analysis are listed in Supplementary Table 3. For both DC and T cell surface staining, 5x10^5^ cells were added to wells of a 96-well U-bottom plate (VWR) and stained with 1-2μl of each antibody resuspended in a total volume of 100μl of 2% FBS in PBS for 30 minutes at 4°C. Subsequent intracellular staining of T cells was performed with the Foxp3 Transcription Factor Staining Buffer Set kit (ThermoFisher) according to the manufacturer’s instructions. After the final washing step, cells were fixed with 2% paraformaldehyde in PBS and acquired on the flow cytometer at ≤2,000 events per second. Data analysis was conducted on 50,000 events per sample with FlowJo software version 10.8.1 (Tree Star Inc.).

### Statistical analysis

For each parameter measured, data were plotted using GraphPad Prism software, version 10.0.2 (GraphPad). Groups were analyzed for normality, then parametric or non-parametric statistical analyses were performed as appropriate. Individual treatments were compared to controls using a Student’s 2 tailed T test or the Mann-Whitney test, depending on normality of the data. Multiple group comparisons were performed using either one-way ANOVA with Tukey’s pairwise comparisons tests for parametric analyses or Kruskal-Wallis with Dunn’s multiple comparisons tests for non-parametric analyses. P values ≤ 0.05 were considered statistically significant.

## Results

### Hookworm-derived proteins reduce *Trypanosoma cruzi*-induced cardiac inflammation

**FIGURE 2:**
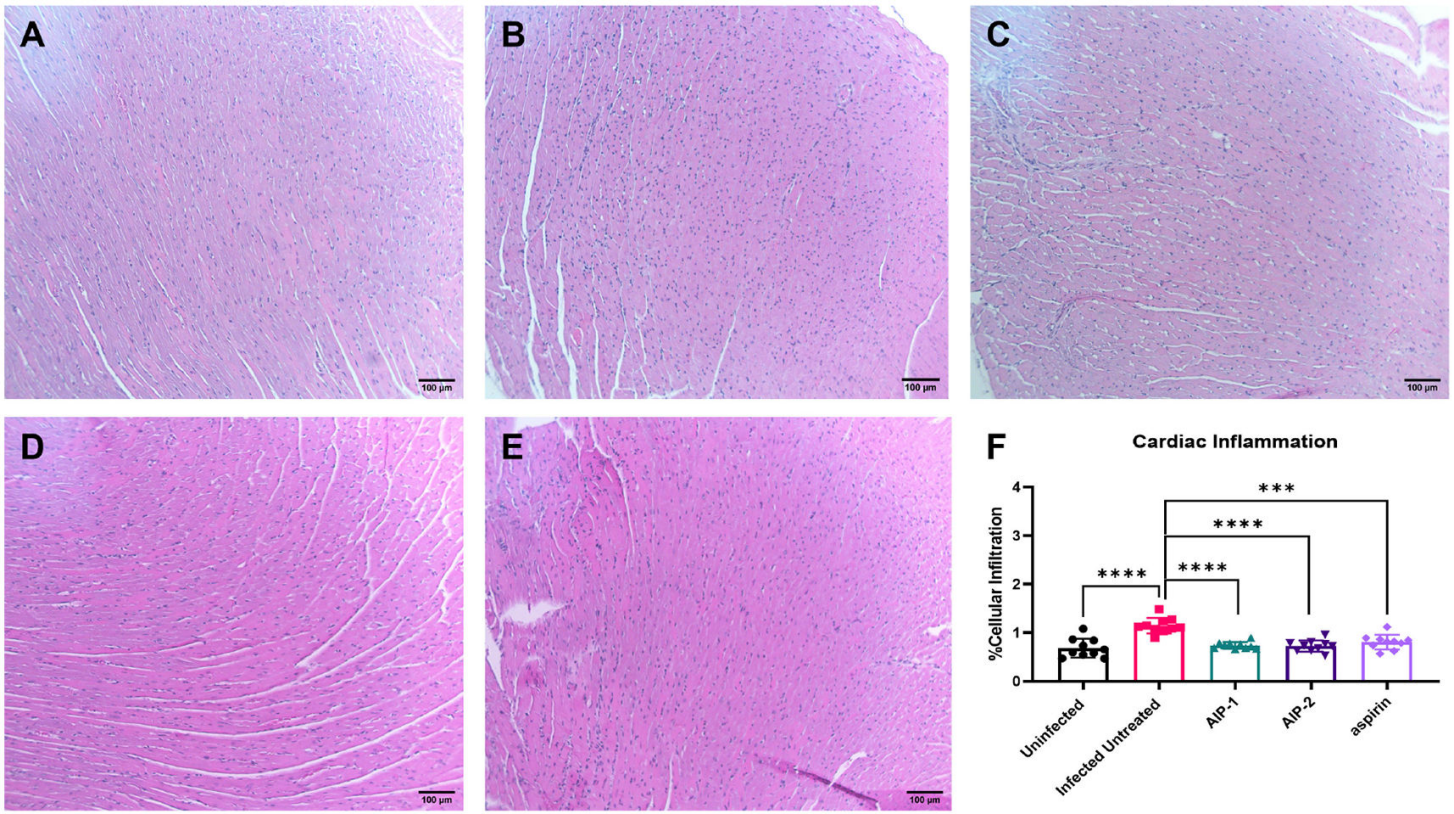
Cardiac cellular infiltrate in heart tissues stained with Hematoxylin and Eosin (H&E). Representative images of left ventricular sections from **(A)** Uninfected **(B)** Infected Untreated **(C)** AIP-1 **(D)** AIP-2 **(E)** aspirin treated mice. Bar = 100μm **(F)** Graph of cellular infiltration in cardiac tissues. Data from individual mice are shown. n=10. Statistical Analysis = Mann-Whitney, p<0.05. Each treatment group is compared to the Infected Untreated group and error bars are defined by Mean with SD. ***p ≤ 0.001; ****p ≤ 0.0001.

**FIGURE 3:**
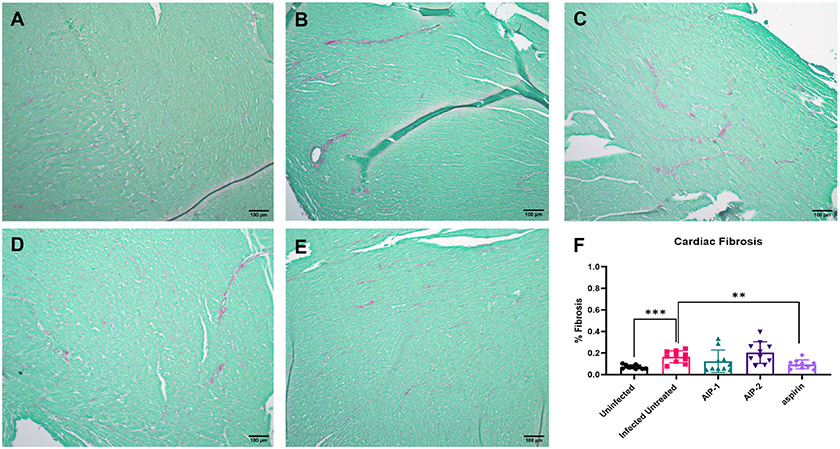
Cardiac fibrosis in heart tissues stained with Picrosirius Red. Representative images of left ventricular sections from **(A)** Uninfected **(B)** Infected Untreated **(C)** AIP-1 **(D)** AIP-2 **(E)** aspirin treated mice. Bar = 100μm **(F)** Graph of the percentage of fibrosis in cardiac tissues. Data from individual mice are shown. n=10 Statistical Analysis = Mann-Whitney, p<0.05. Each treatment group is compared to the Infected Untreated group and error bars are defined by Mean with SD. **p ≤ 0.01; ***p ≤ 0.001.

To investigate the impact of the hookworm derived proteins AIP-1 and AIP-2 on *T. cruzi*-induced cardiac inflammation, we quantified the infiltration of leukocytes into cardiac tissue in representative H&E-stained sections of the left ventricle after treatment. Figures 2A, B, F) shows that mice that were infected and left untreated had significantly increased infiltration of leukocytes compared to age-matched uninfected mice. However, infected mice that were treated with either AIP-1 (Figures 2C, F) or AIP-2 (Figures 2D, F) had significantly reduced infiltration of leukocytes into cardiac tissue compared to infected untreated controls. As expected, infected mice treated with the anti-inflammatory drug aspirin also had significantly reduced infiltration of leukocytes into cardiac tissue (Figures 2E, F). Chronic infection with *T. cruzi* also induces significant fibrosis in cardiac tissues, as evidenced by the accumulation of collagen fibers (Rossi et al., 2003). Therefore, we also quantified collagen deposition in representative picrosirius red stained sections of left ventricles in chronically infected mice after treatment. Figure 3 shows that mice that were infected and left untreated had significantly increased collagen deposition compared to age-matched uninfected mice (Figures 3A, B, F). Treatment with either AIP-1 or AIP-2 did not significantly impact the level of collagen deposition induced by chronic *T. cruzi* infection (Figures 3C, D, F). However, aspirin treatment did significantly reduce collagen deposition compared to infected untreated controls (Figures 3E, F). Collectively these data suggest that AIP-1 and AIP-2 treatment reduces infiltration of inflammatory cells into cardiac tissue, modulating the inflammatory response and partially ameliorating the pathology induced by chronic *T. cruzi* infection.

### Hookworm-derived proteins modulate *Trypanosoma cruzi* induced inflammatory responses

**FIGURE 4:**
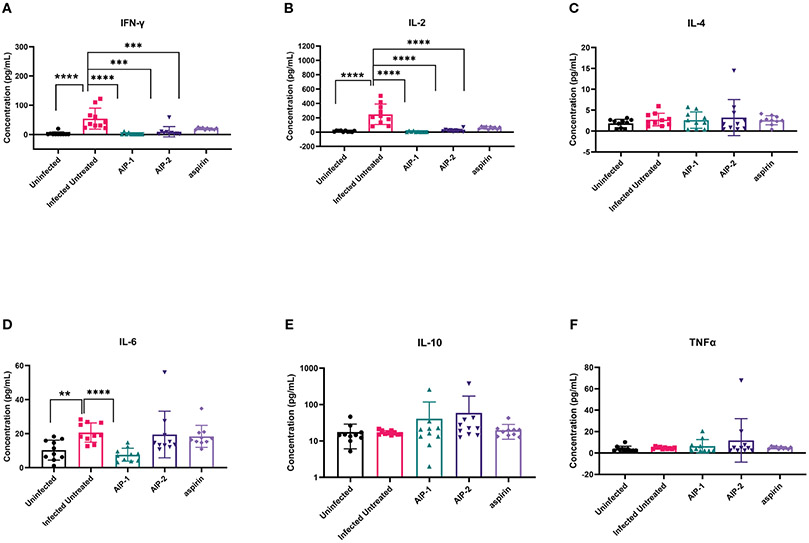
Pro- and anti-inflammatory cytokines measured in cardiac tissue lysate. The concentration of **(A)** IFNγ, **(B)** IL-2, **(C)** IL-4, **(D)** IL-6, **(E)** IL-10, and **(F)** TNFα were measured by Luminex. Data from individual mice are shown. n=10. Statistical Analysis = Mann-Whitney, p<0.05. Each treatment group is compared to the Infected Untreated group and error bars are defined by Mean with SD. **p ≤ 0.01; ***p ≤ 0.001; ****p ≤ 0.0001.

**FIGURE 5:**
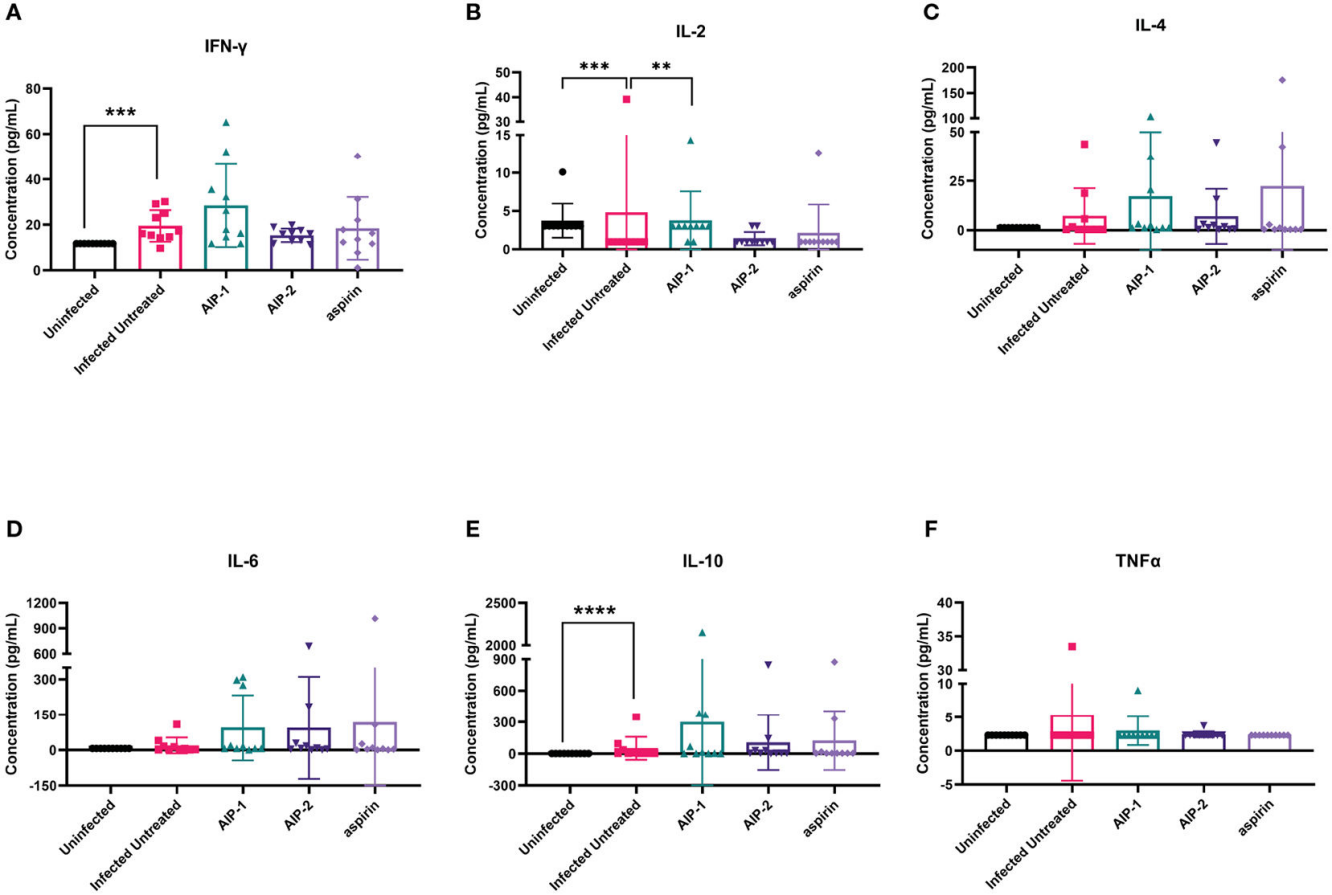
Pro- and anti-inflammatory cytokines measured in serum. The concentration of **(A)** IFNγ, **(B)** IL-2, **(C)** IL-4, **(D)** IL-6, **(E)** IL-10, and **(F)** TNFα were measured by Luminex. Data from individual mice are shown. n=10. Statistical Analysis = Mann-Whitney, p<0.05. Each treatment group is compared to the Infected Untreated group and error bars are defined by Mean with SD. **p ≤ 0.01; ***p ≤ 0.001; ****p ≤ 0.0001.

Next, we quantified the cardiac inflammatory response modulated by AIP-1 and AIP-2 treatment by measuring levels of cytokines in cardiac tissue lysates. Figure 4 shows that infected untreated mice had significantly elevated levels of IFNγ, IL-2 and IL-6 (Figures 4A, B, D, respectively) when compared to age-matched uninfected controls. Importantly, treatment with AIP-1 significantly reduced levels of IFNγ, IL-2 and IL-6 (Figures 4A, B, D, respectively), while treatment with AIP-2 also significantly reduced levels of IFNγ and IL-2 (Figures 4A, B, respectively). Similarly, aspirin treatment significantly reduced levels of IFNγ and IL-2 (Figures 4A, B, respectively). Cardiac levels of IL-4, IL-10 and TNFα were not significantly affected by infection or treatment (Figures 4C, E, F, respectively). To compare tissue-specific levels of cytokines to circulating cytokines, we measured cytokine levels in the serum. As shown in Figure 5, serum levels of IFNγ and IL-10 (Figures 5A, E, respectively) were significantly elevated in infected untreated mice when compared to age-matched uninfected controls, whereas levels of IL-2 were significantly decreased in infected untreated mice (Figure 5B). Treatment with AIP-1 significantly increased serum levels of IL-2 (Figure 5B) when compared to infected untreated mice, however there were no other differences in serum levels of IFNγ, IL-4, IL-6, IL-10 or TNFα resulting from any treatment. We also measured AIP-1 and AIP-2 specific serum IgG levels. The results show that mice intraperitoneally administered with AIP-1 produced limited IgG antibody in their sera, however, mice that received AIP-2 produced higher level of IgG antibody than mice which received AIP-1 (Supplemental Figure 1). The results indicate AIP-2 possesses stronger immunogenicity than AIP-1, possibly because the yeast-expressed AIP-2 is highly glycosylated. There was no cross-reaction between AIP-1 and AIP-2 observed in the serological ELISA results.

**FIGURE 6:**
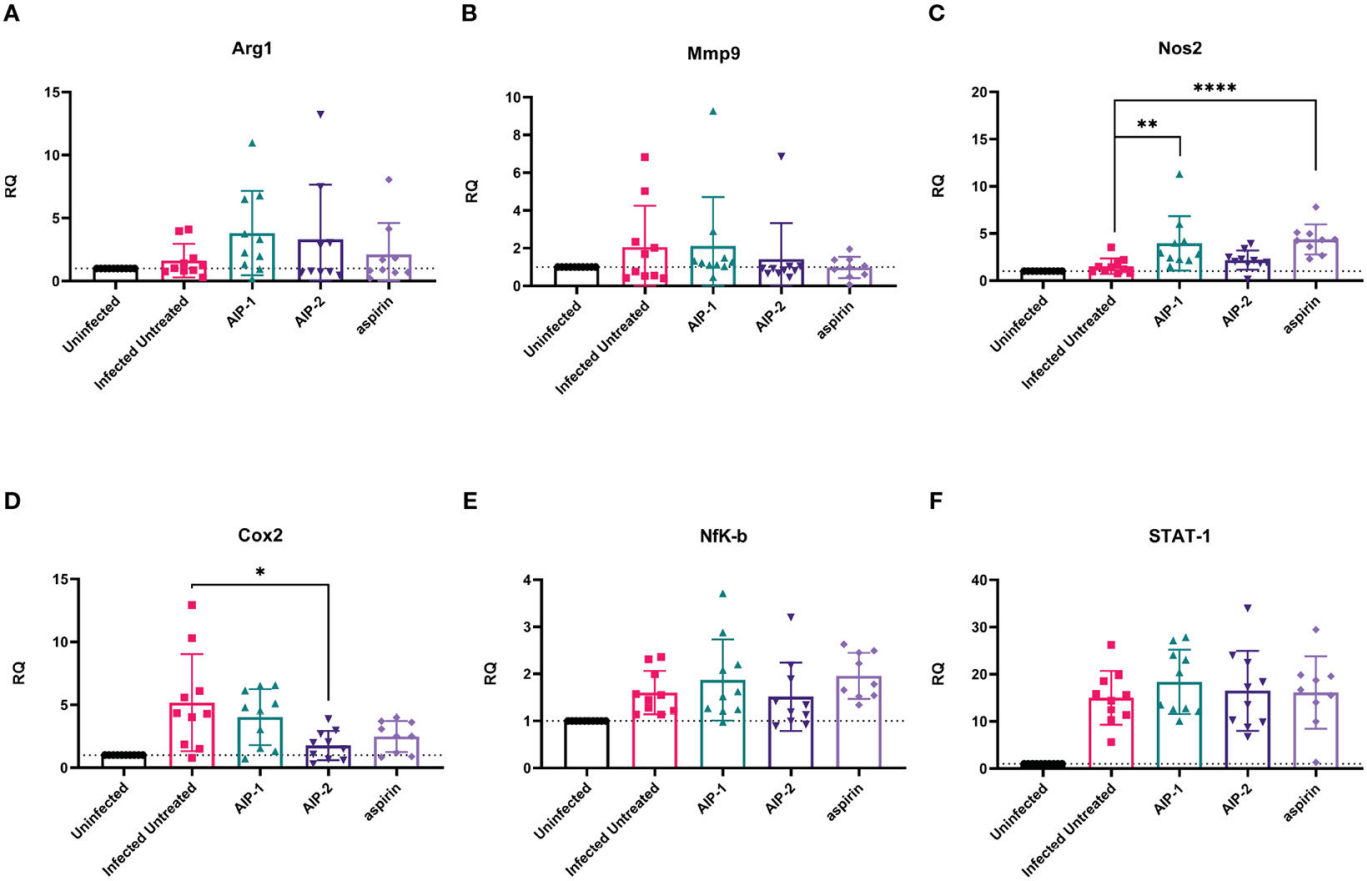
Relative quantification of gene expression levels in cardiac tissues. Expression of **(A)** Arg1, **(B)** Mmp9, **(C)** Nos2, **(D)** Cox2, **(E)** NFκB, and **(F)** STAT-1 mRNA levels were measured from cardiac tissues by RT-PCR. Data from individual mice are shown. n=10. Statistical Analysis = Mann-Whitney, p<0.05. Each treatment group is compared to the Infected Untreated group and error bars are defined by Mean with SD. *p ≤ 0.05; **p ≤ 0.01; ****p ≤ 0.0001.

**FIGURE 7:**
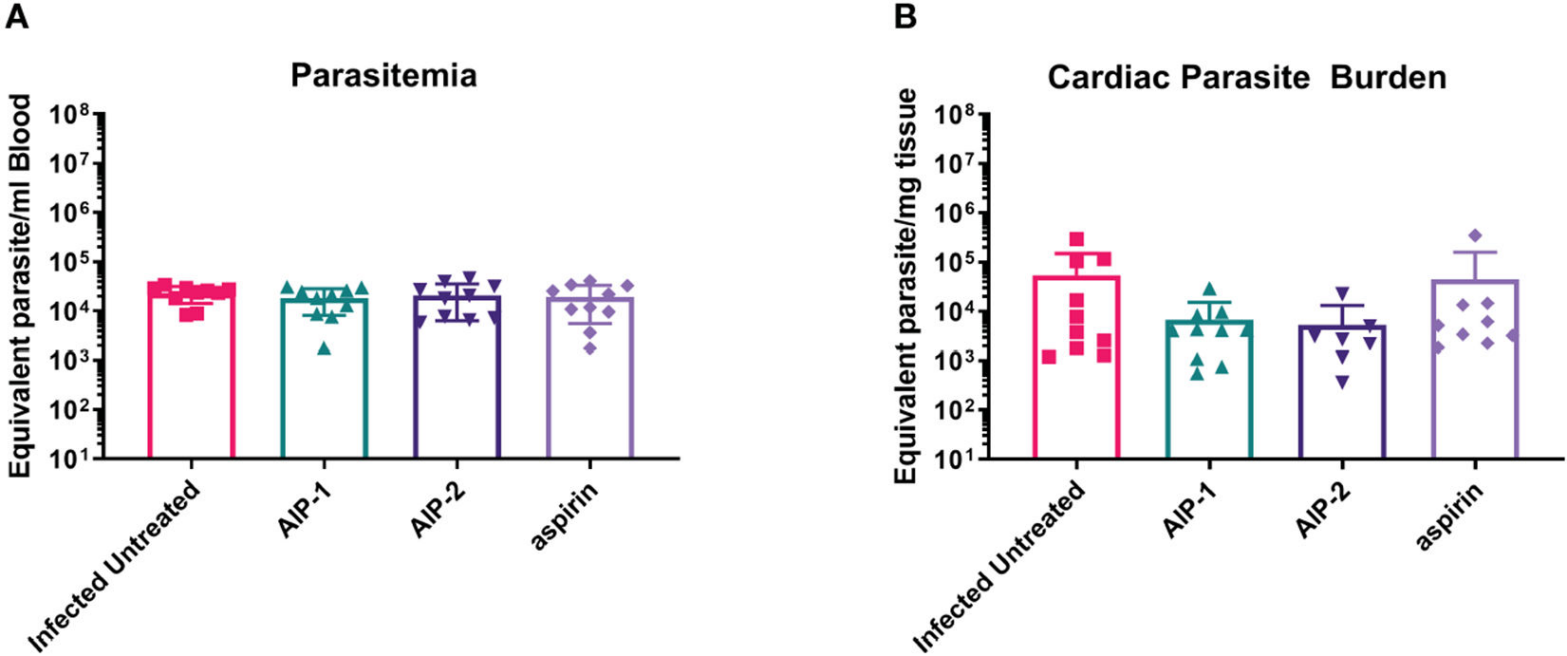
Parasite burdens. Parasite burdens were measured at the end of the study in **(A)** blood and **(B)** cardiac tissue by quantitative PCR (qPCR). Data from individual mice are shown. n=10. Statistical Analysis, Mann-Whitney; p<0.05. Error bars are defined by Mean with SD.

Finally, we quantified the expression of several inflammatory genes known to be upregulated in the cardiac tissue of mice experimentally infected with *T. cruzi*, including Arg1, MMP9, NOS2, COX-2, NFκB and STAT1 (Corral et al., 2013; Wan et al., 2016; Hoffman et al., 2021). Figure 6 shows that while there was no difference in the expression of Arg1 and Mmp9 (Figures 6A, B, respectively), expression of NOS2 (iNOS) was significantly increased in mice treated with AIP-1 or aspirin compared to infected untreated controls (Figure 6C). In contrast, expression of COX-2 was significantly reduced by treatment with AIP-2 (Figure 6D). Neither treatment with AIP-1 nor AIP-2 significantly impacted the expression of NFκB (Figure 6E) or STAT1 (Figure 6F). Together, these data suggest that AIP-1 and AIP-2 may modulate *T. cruzi*-induced inflammatory responses by regulating the expression of inflammatory genes, specifically NOS2 and COX-2. Altogether, these data show that chronic infection with *T. cruzi* significantly increases cardiac inflammatory responses which are modulated by treatment with either AIP-1 or AIP-2. These effects were seen without any significant reduction of cardiac or circulating parasite burdens (Figures 7A, B). This suggests that AIP-1 and AIP-2 treatment directly modulate host inflammatory responses in a mouse model of chronic *T. cruzi* infection, similar to what has previously been demonstrated in mouse models of allergic asthma and inflammatory bowel disease (Navarro et al., 2016; Ferreira et al., 2017; Buitrago et al., 2021).

### Hookworm protein pulsed DC prime T cells towards a regulatory phenotype

To assess the role of hookworm-derived proteins AIP-1 and AIP-2 on the immune modulation of innate antigen presenting cells, and to evaluate the resulting phenotypic changes, bone marrow-derived immature DC were loaded with AIP-1 (AIP-1 DC) and AIP-2 (AIP-2 DC) proteins and compared with control DC and DCs loaded with *T. cruzi* parasite lysate (*T. cruzi* DC). Control DC were not loaded with *T. cruzi* lysate, AIP-1 or AIP-2 proteins but were matured similarly with pro-inflammatory cytokine cocktail. The gating strategy for the flow cytometry analysis is indicated with FSC/SSC and single-cell gates (Supplemental Figure 2A) and representative flow plots are shown (Supplementary Figure 3). Results indicated that compared to the control or *T. cruzi* DC, there was no significant upregulation in the CD11c+CD11b+ subset among AIP-1 DC and AIP-2 DC (Supplemental Figure 2B). Similarly, there was no significant difference in the expression of maturation markers (CD80+CD86+) between the groups (Supplemental Figure 2C). However, although not significant, there was a slight increase in the MHC II and Signal Regulatory Protein α expression (MHCII+SIRPα+) levels within the CD11c+CD11b+ subset (Supplemental Figure 2D) indicative of a slight enhancement in the immunoregulatory phenotype. While inflammatory DC prime cytotoxic T cells and generate T_H_1-skewed pro-inflammatory immune responses, immunoregulatory DC dampen T_H_1 responses and prime regulatory T cell responses. These results indicate that hookworm proteins AIP-1 and AIP-2 modulate the antigen-presenting DC slightly towards an immunoregulatory phenotype.

**FIGURE 8:**
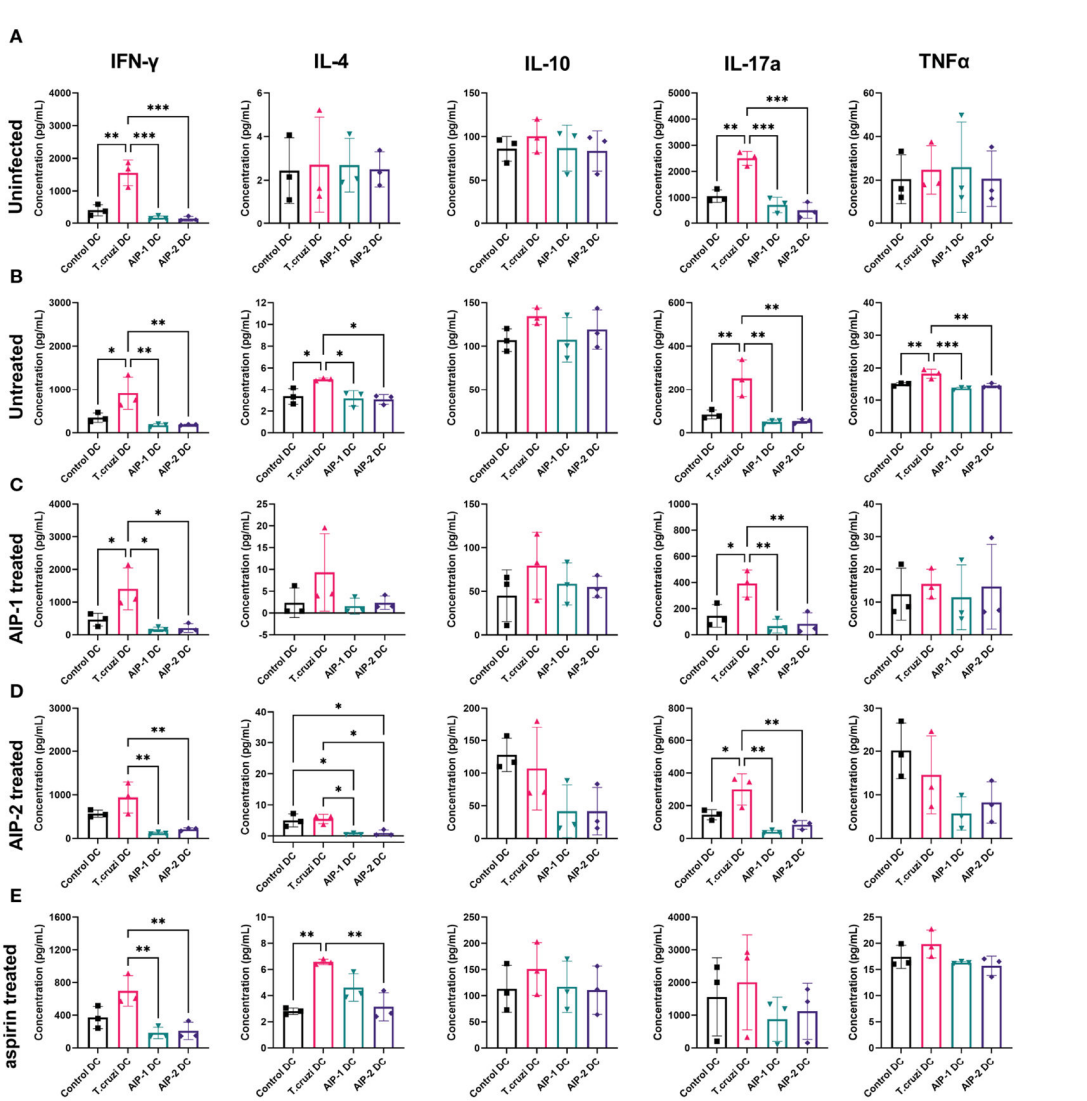
Secreted cytokines from in vitro restimulated splenocytes. Splenocytes from uninfected **(A)**, Infected untreated **(B)**, AIP-1 treated **(C)**, AIP-2 treated **(D)**, and aspirin treated **(E)** mice were harvested, and co-cultured with DC incubated with AIP-1, AIP-2 proteins or *T. cruzi* lysate. 48-hour culture supernatants were then evaluated for pro and anti-inflammatory cytokines (IFN-γ, IL-4, IL-10, IL-17a TNFα,) by Luminex analysis. Statistical analyses was performed using ANOVA with Tukey′s multiple comparison test comparing the mean of each column to the mean of every other column *p ≤ 0.05; **p ≤ 0.01; ***p ≤ 0.001. Error bars are defined by Mean with SD.

To determine the effect of hookworm protein antigen presentation by DC on downstream T cell responses, AIP-1 DC, AIP-2 DC, and *T. cruzi* DC were incubated with splenocytes isolated from uninfected, infected untreated, AIP-1 treated, AIP-2 treated, and aspirin treated mice. Splenocytes from the treatment groups were also incubated with control DC for background levels of DC-mediated stimulation. Culture supernatants were analyzed after 48 hours of incubation and assayed for inflammatory cytokines. As seen from Figure 8A, in the uninfected group, compared to incubation with control DC and *T. cruzi* DC, incubation with AIP-1 DC and AIP-2 DC resulted in significantly lower levels of secretion of pro-inflammatory cytokines like IFNγ, and IL-17A. Similar results were seen in the infected-untreated, AIP-1 treated and AIP-2 treated (Figures 8B-D respectively) groups. In the aspirin treated group, there was a significantly lower level of secretion of IFNγ and IL-4 (Figure 8E). Significantly lower levels of IL-4 secretion were also seen among the untreated and AIP-2 treated groups while lower levels of TNFα were seen in the untreated group (Figures 8B, D, respectively). However, there was no discernable difference in the levels of IL-10 among the different treatment groups. Overall, the results indicated that compared to the stimulation with control DC and *T. cruzi* DC, stimulation with AIP-1 DC or AIP-2 DC resulted in significantly lower levels of pro-inflammatory cytokine production highlighting the anti-inflammatory responses meditated by the hookworm proteins.

**FIGURE 9:**
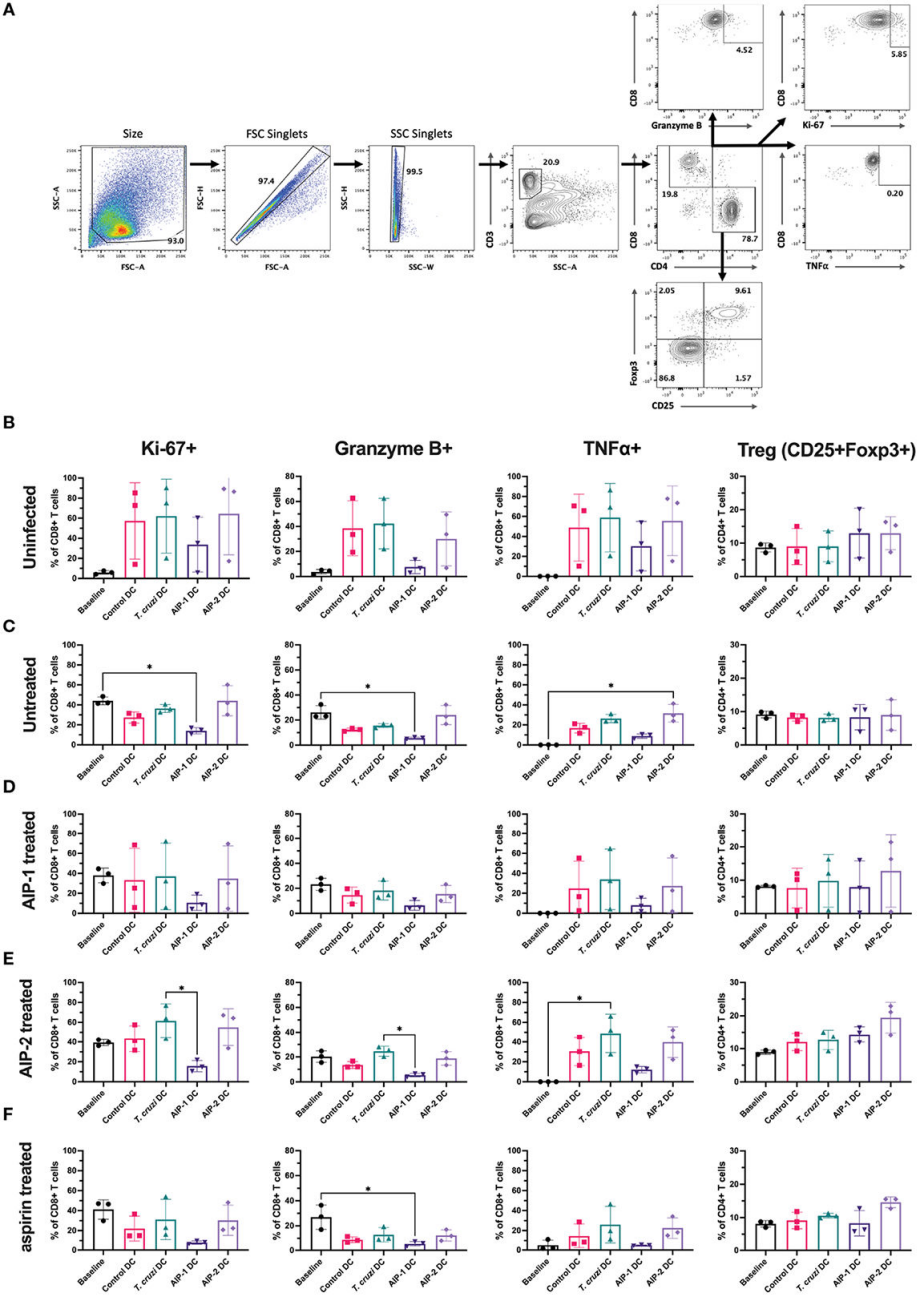
Immunophenotype of T cells before and after co-culturing splenocytes with DC. Splenocytes harvested from uninfected-untreated, infected-untreated, AIP-1 treated, AIP-2 treated, or aspirin-treated mice were co-cultured with DC loaded with AIP-1 protein, AIP-2 protein, *T. cruzi* lysate, or no protein (control DC). Flow cytometry analysis was performed on pre-co-culture, unstimulated splenocytes (baseline) and DC-stimulated splenocytes after 13 days of co-culture. The overall gating strategy for flow cytometry analysis is shown in **(A)**. Splenocytes were analyzed for proliferation (CD8+KI67+), cytotoxicity (CD8+GranzymeB+), inflammation (CD8+TNFα+) and regulatory phenotypes (CD4+CD25+Foxp3+). Comparisons were made between all groups among uninfected untreated control mice **(B)**, infected untreated mice **(C)**, infected AIP-1 treated mice **(D)**, infected AIP-2 treated mice **(E)** and infected aspirin treated mice **(F)**. Statistical analyses was performed using Kruskal-Wallis with Dunn’s multiple comparisons tests comparing the mean rank of each column to the mean rank of every other column. Error bars are defined by mean with SD. * p<0.05.

Further, T cells stimulated with the DC were phenotyped after 13 days of co-culture and compared with the baseline levels prior to DC stimulation. Splenocytes were gated to represent the T cell population (Figure 9A) and representative flow plots are shown (Supplemental Figures 4-7). As shown in Figure 9B, in the uninfected group, compared to the pre co-culture (Day 0) baseline levels, stimulation with DC groups (control DC, *T. cruzi* DC, AIP-1 DC and AIP-2 DC) resulted in a trend towards elevated levels of proliferation (Ki67+), cytotoxicity (Gzm+) and inflammation markers (TNFα+) within the CD8+ T cell population. However, compared to stimulation with either control DC, *T. cruzi* DC or AIP-2 DC, stimulation with AIP-1 DC resulted in a trend towards down regulation of proliferating, cytotoxic and pro-inflammatory T cells suggesting the anti-inflammatory role of the AIP-1 hookworm protein. Also, there was a trend towards elevation of CD4+CD25+Foxp3+ T cells with both AIP-1 DC and AIP-2 DC stimulation compared to the baseline or stimulation with control DC and *T. cruzi* DC, suggesting the role of the hookworm proteins in skewing a regulatory T cell phenotype. A similar trend was seen in the untreated (Figure 9C), AIP-1 treated (Figure 9D), AIP-2 treated (Figure 9E) and aspirin treated (Figure 9F) groups, where stimulation with AIP-1 DC resulted in a trend towards skewing an anti-inflammatory response and stimulation with AIP-1 DC and AIP-2 DC resulted in a trend skewing towards a regulatory T cell phenotype. In the untreated group, stimulation with AIP-1DC resulted in significant downregulation of proliferation and cytotoxicity markers while stimulation with AIP-2 DC resulted in elevated inflammation markers Granzyme B and TNFα in the CD8+ T cell population (Figure 9C). These results suggest that the two hookworm proteins AIP-1 and AIP-2 might act through different pathways to generate a regulatory phenotype that is not skewed towards pro-inflammatory T_H_1 responses. While AIP-1 in general showed a tendency towards slightly dampened pro-inflammatory response, AIP-2 showed a tendency towards slightly elevated regulatory phenotype. The precise mechanisms through which these two proteins mediate immunoregulation need further investigation using a larger sample size.

## Discussion

Here we investigated the immunomodulatory effects of two recombinant hookworm-secreted proteins, AIP-1 and AIP-2, on inflammatory responses in a mouse model of chronic *T. cruzi* infection. Both AIP-1 and AIP-2 significantly reduced cellular infiltrate into cardiac tissue, suggesting reduced inflammation similar to what was previously observed with AIP-1 treatment in mouse models of colitis and with AIP-2 treatment in mouse models of allergic airway disease (Navarro et al., 2016; Ferreira et al., 2017; Buitrago et al., 2021). Concurrent with reduced cardiac cellular infiltrate, both AIP-1 and AIP-2 significantly reduced cardiac levels of IFNγ, while AIP-1 also significantly reduced cardiac IL-6 levels. Serum levels of both IFNγ and IL-6 are significantly elevated in patients with CCC and correlate with disease severity (López et al., 2006; Keating et al., 2015). Thus, the impact of both AIP-1 and AIP-2 on reducing inflammatory cytokine levels in cardiac tissues suggests that these proteins may ameliorate clinical symptoms of cardiac disease. In addition to measuring cytokine levels in tissue, we measured cardiac expression of key inflammatory markers by RT-PCR. Both AIP-1 and aspirin increased the expression of NOS2 in cardiac tissue (Figure 6). NOS2, the gene for inducible Nitric Oxide Synthase (iNOS), is a key signaling molecule in the induction of NO. NO has been shown to have both beneficial and detrimental effects on *T. cruzi* infection (Fresno and Gironès, 2021). NO activates macrophage-killing mechanisms and can be protective against *T. cruzi* infection (Muñoz-Fernández et al., 1992; Lee et al., 2017). In fact, mice defective in iNOS signaling are unable to control *T. cruzi* parasite burdens and succumb to infection (Hölscher et al., 1998). However, it has also been shown that myeloid-derived suppressor CD11b+Ly6C+Ly6G^lo^ cells (MDSC) are present in the spleens and hearts of *T. cruzi*-infected mice, they express iNOS and Arg1, and they induce expansion of Foxp3-expressing regulatory T cells (Huang et al., 2006; Fresno and Gironès, 2021). Further, induction of NO by iNOS interferes with IL-2R signaling and alters the stability of IL-2 mRNA (Bronte and Zanovello, 2005). In our model, we showed that AIP-1 treatment increased cardiac NOS2 expression (Figure 6C), which could be due to expression in cardiac MDSC. Further, AIP-1 treatment significantly reduced IL-2 in cardiac tissue (Figure 4B). Thus, the effects of AIP-1 on *T. cruzi* inflammatory responses may be due to the combined effects of inducing MDSC which may play a role in the reduced number of cardiac infiltrating leukocytes. Additionally, we found that AIP-2 treatment significantly decreased the expression of COX-2 (Figure 6D). Cyclooxygenase (COX) enzymatic activity converts arachidonic acid to various lipids, including prostaglandins and thromboxanes, which mediate inflammatory processes (Dubois et al., 1998). Increased cardiac expression of COX-2 has been observed in mice acutely infected with *T. cruzi* (Corral et al., 2013). Additionally, thromboxane A2 (TXA2) is increased in the plasma of chronically infected mice, where it mediates cardiac tissue damage by exacerbating myocyte apoptosis and enhancing cardiac dysfunction (Cardoni and Antunez, 2004; MaChado et al., 2011). Further, treatment of chronically infected mice with the COX inhibitor acetylsalicylic acid (aspirin) reduced levels of circulating prostaglandins and improved cardiac function (Mukherjee et al., 2011). Wild type mice acutely infected with *T. cruzi* had COX-2 expressing CD11b+ cells that infiltrate cardiac tissue, whereas mice deficient in COX-2 signaling had reduced cardiac infiltration of macrophages and DC, as well as reduced expression of inflammatory cytokines IL-6, IFNγ, and TNFα compared to wild type mice, demonstrating a key role for COX-2 signaling in *T. cruzi*-induced myocarditis mediated by CD11b+ cells (Guerrero et al., 2015). Our data corroborate the *T. cruzi*-induced inflammatory mediators in cardiac tissue, including increased levels of cardiac inflammatory cytokines IL-6 and IFNγ as well as increased expression of NOS-2 and COX-2. Importantly, we presented evidence that the hookworm-derived proteins AIP-1 and AIP-2 modulate cardiac inflammatory responses in NOS2 and COX2-dependent manners, respectively. Our data suggest that AIP-1 and AIP-2 may ameliorate *T. cruzi*-induced cardiac dysfunction, and this will be investigated in future studies.

The immunomodulatory role of hookworm-derived proteins in chronic inflammation has been studied in the past. In colitis and asthma, reduced inflammation by hookworm proteins was mediated by tolerogenic CD11c+ cells, reduced levels of inflammatory cytokines and upregulation of regulatory T cells. Dendritic cells (DC) are one of the key immune cell types involved in both innate and adaptive immune responses, by initially phagocytosing pathogens at infection sites to control the spread, and subsequently stimulating lymphocytes in immune tissues, such as spleen and lymph nodes, to initiate development of the adaptive immune response (Sheel and Engwerda, 2012; Dos-Santos et al., 2016; Ginhoux and Guilliams, 2016; Cerbán et al., 2020). During innate immune responses, DC upregulate iNOS and subsequently NO production in response to inflammatory stimuli, resulting in pathogen killing (Lee et al., 2017; Thwe and Amiel, 2018). Additionally, DC are the most efficient antigen-presenting cell (APC) for initiating adaptive immune response. Within immune tissues, DC can be polarized to generate a pro-inflammatory T_H_1 response where they present antigen to naïve CD8+ T cells on MHC Class I molecules and to naïve CD4+ T cells on MHC Class II molecules, simultaneously providing costimulatory molecules CD80 and CD86, and secreting pro-inflammatory cytokines, such as IL-1β, IL-6, TNFα, and IL-12, which are critical to inducing activation and proliferation of naïve T cells into antigen-specific effector cells (Blum et al., 2013; Gil-Jaramillo et al., 2016). While T_H_1 polarization of DC induces an inflammatory response, it is the tolerogenic regulatory DC that dampen the inflammation-induced tissue damage by stimulating regulatory T cells. In this study, we investigated the regulatory effects of hookworm-derived proteins on dendritic cell phenotype and the subsequent effect on downstream T cell function. We have demonstrated that compared to incubation with T. cruzi protein lysate, incubation with hookworm proteins AIP-1 and AIP-2 induced a slight upregulation of CD11c+CD11b+MHCII+SIRPα+ cells indicative of an immunoregulatory phenotype (Supplemental Figure 2D). The CD11b+CD11c+ DC subset has earlier been reported to play a role in generating tolerogenic and regulatory responses against chronic inflammation and hence is exploited to treat autoimmune diseases (Li et al., 2008). SIRPα is expressed by macrophages and DC and upon binding to CD47, sending an inhibitory signal to the phagocytic cells that results in inefficient phagocytosis (Willingham et al., 2012). Anti-SIRPα antibodies that target CD47-SIRPα axis have been reported to show promise in promoting anti-tumor immunity (Wu et al., 2022). The slight upregulation of this phenotype (MHCII+SIRPα+CD11c+CD11b +) is suggestive of immunoregulatory DC that mediate regulatory T cell responses. Increasing the antigenic incubation time from 3 hours to 6-16 hours as reported by other studies might result in a significant upregulation of this phenotype compared to the relatively slight upregulation that was seen in the current study (Cuellar et al., 2009). Further, when these DC were used to stimulate splenocytes derived from the uninfected/infected and treated mice, there was a significant down regulation of inflammatory cytokine secretion in all the treated groups with AIP-1 DC and AIP-2 DC stimulation (Figure 8). T cell phenotypic changes were also evident especially in the CD8 compartment where stimulation with AIP-1 DC resulted in a downregulation of proliferating, cytotoxic and pro-inflammatory CD8 T cells (Figure 9). However, stimulation with both AIP-1 DC and AIP-2 DC seemed to have a modest effect on the regulatory compartment of CD4+ CD25+Foxp3+ T cells (Figure 9). Similar results have been reported in the past where treatment of DC with hookworm-derived metalloprotease inhibitors has resulted in a tolerogenic DC phenotype and generation of regulatory and suppressive T cells (Cuellar et al., 2009). In a mouse model of allergic asthma, suppression of airway inflammation was dependent on both mesenteric CD103^+^DC and Foxp3^+^ regulatory T cells (Tregs). AIP-2 induced expansion of mesenteric lymph node DC resulting in the generation of Tregs that homed to the mucosa promoting long-term protection against allergic asthma (Navarro et al., 2016). It is possible that the hookworm-derived proteins AIP-1 and AIP-2 act through independent pathways to dampen T_H_1 responses and promote regulatory responses.

Hookworm-secreted AIP-1 and AIP-2 share structural homology with tissue inhibitor of matrix metalloproteases (TIMPs) and are known to inhibit the function of some human MMP (Zhan et al., 2008; Cuellar et al., 2009). Activated DC and macrophages upregulate MMPs to launch an inflammatory response and inhibition of MMPs has been a therapeutic option for chronic conditions. Blocking MMP-13 in DC has been shown to reduce the surface MHC-I expression, and antigen presentation, resulting in changes to the cytokine/chemokine profile of DC and subsequent reduction in CD8+ T cell activation (Bartmann et al., 2016). In a mouse model of collagen-induced arthritis (CIA), inhibition of MMP-9 significantly blocked DC trafficking and delayed CIA (He et al., 2018). In another study, hookworm protein Na-AIP-1 significantly suppressed CIA when used either as a monotherapy or in combination with methotrexate (MTX) (Langdon et al., 2022). However, the precise mechanisms through which specific hookworm-derived proteins inhibit the various MMPs and the specific interactions mediating their function is not clearly understood and need further investigation. Further, several studies suggest that the immunoregulatory phenotype of DC leading to downstream regulatory T cell responses depends upon TGFβ signaling. The role of TGFβ in generating this immunoregulatory phenotype has not been explored in the current study. Overall, the current proof of principle study demonstrates that hookworm-derived proteins mediate an immunoregulatory effect via modulating DC and T cell responses, in a mouse model of CCC. However, the specific and precise mechanisms through which the hookworm-derived proteins induce DC-mediated immunoregulatory phenotype needs further investigation.

In this study, we present strong evidence for the beneficial immunomodulatory effect of hookworm derived anti-inflammatory proteins on CCC. However, some study limitations were acknowledged and are under careful consideration for future evaluation. In this study, we evaluated endpoint cardiac pathology 14 days after treatments were initiated to determine the efficacy of AIP-1 and AIP-2 on improving cardiac health. While this study design allowed us to determine the immediate treatment effects, a longer timeline in future studies will be necessary to determine the duration of treatment effects. We showed that both AIP-1 and AIP-2 reduced cardiac cellular infiltrate, and in future studies identification and quantification of CD4+ and CD8+ T cells, as well as CD4+CD25+Foxp3+ cells, will better characterize the inflammatory cell infiltrate and determine the correlation between tissue-specific T cell responses and peripheral T cell responses evaluated from splenocytes. Further, while we showed that both AIP-1 and AIP-2 significantly reduced cardiac inflammation, there was no apparent effect on cardiac fibrosis, which is another key pathology of CCC (Rossi et al., 2003). Cardiac fibrosis is progressive in mouse models of chronic infection (Hoffman et al., 2019), thus evaluating tissue pathology at later times after infection and treatment will reveal if AIP-1 and AIP-2 treatment results in immediate reduction of inflammation, and later reduction of fibrosis. We evaluated the impact of AIP-1 and AIP-2 treatments in a female mouse model of chronic *T. cruzi* infection. However, male sex is a risk factor for more severe CCC in humans (Basquiera et al., 2003; Sabino et al., 2013), thus evaluation of treatment in both male and female mice will be necessary to determine the extent of treatment efficacy. Our results also showed that both AIP-1 and AIP-2 induced skewing towards regulatory BMDC which stimulated slightly elevated CD4+CD25+Foxp3+ splenocytes, agreeing with previously published results (Cuellar et al., 2009). Future studies with longer antigen incubation times are warranted. Quantification of regulatory cytokines, including IL-10 and TGFβ, specifically from AIP-1 and AIP-2 treated BMDC will further define the immunomodulatory effects of these proteins in our model of chronic *T. cruzi* infection. Further, alternatively activated M2 macrophages are another key source of IL-10 and TGFβ and serve to regulate tissue damage (Mantovani et al., 2013; Shapouri-Moghaddam et al., 2018). Thus, future studies to specifically define the impact of AIP-1 and AIP-2 on macrophages and cytokine production will better define the mechanisms of efficacy for these proteins.

Patients with CCC have higher levels of cardiac inflammation than Chagas patients with mild disease and asymptomatic patients, and ultimately have a worse prognosis than patients with non-inflammatory cardiomyopathies (Higuchi et al., 1987; Bestetti and Muccillo, 1997). Unfortunately, only two antiparasitic drugs are licensed to treat infection, benznidazole and nifurtimox, and both have prolonged treatment courses and induce significant side effects, resulting in many patients terminating treatment early (Viotti et al., 2009; Jackson et al., 2010; Urbina, 2010; Molina et al., 2015). Further, anti-parasitic treatment does not address the underlying deleterious host responses that lead to clinical disease and cardiac death (Morillo et al., 2015). Therefore, improved treatment strategies, and specifically strategies that modulate host responses, are urgently needed. The data presented here suggest that treatments that reduce host inflammatory responses, such as hookworm-derived anti-inflammatory proteins, are an important tool for reducing cardiac pathology, and may serve to improve clinical outcomes for the almost 7 million individuals with Chagas disease globally (Disease et al., 2016; GBD 2016 Disease and Injury Incidence and Prevalence Collaborators, 2017).

## Supplementary Material

Supplementary material

## Data Availability

The datasets presented in this study can be found in online repositories. The names of the repository/repositories and accession number(s) can be found in the article/Supplementary Material.

## References

[R1] Abrahamsohn IA, and Coffman RL (1996). Trypanosoma cruzi: IL-10, TNF, IFN-gamma, and IL-12 regulate innate and acquired immunity to infection. Exp. Parasitol 84, 231–244. doi: 10.1006/expr.1996.0109 8932773

[R2] Aliberti JC, Cardoso MA, Martins GA, Gazzinelli RT, Vieira LQ, and Silva JS (1996). Interleukin-12 mediates resistance to Trypanosoma cruzi in mice and is produced by murine macrophages in response to live trypomastigotes. Infect. Immun 64, 1961–1967. doi: 10.1128/iai.64.6.1961-1967.1996 8675294 PMC174023

[R3] Almeida GG, Rimkute I, do Vale INPC., Liechti T, Henriques PM, Roffe E, (2022). Chagasic cardiomyopathy is marked by a unique signature of activated CD4(+) T cells. J. Transl. Med 20, 551. doi: 10.1186/s12967-022-03761-5 36447264 PMC9708147

[R4] Alvarez JM, Borges da Silva H, Marinho CR, Bortoluci KR, Sardinha LR, (2014). Chagas disease: still many unsolved issues. Mediat. Inflammation 2014, 912965. doi: 10.1155/2014/912965 PMC4101227 25104883

[R5] Bartmann J, Frankenberger M, Neurohr C, Eickelberg O, Noessner E, and Wulffen WV (2016). A novel role of MMP-13 for murine DC function: its inhibition dampens T-cell activation. Int. Immunol 28, 473–487. doi: 10.1093/intimm/dxw008 26921214

[R6] Basquiera AL, Sembaj A, Aguerri AM, Omelianiuk M, Guzman S, Moreno Barral J, (2003). Risk progression to chronic Chagas cardiomyopathy: influence of male sex and of parasitaemia detected by polymerase chain reaction. Heart 89, 1186–1190. doi: 10.1136/heart.89.10.1186 12975414 PMC1767891

[R7] Bestetti RB, and Muccillo G (1997). Clinical course of Chagas’ heart disease: a comparison with dilated cardiomyopathy. Int. J. Cardiol 60, 187–193. doi: 10.1016/S0167-5273(97)00083-1 9226290

[R8] Blum JS, Wearsch PA, and Cresswell P (2013). Pathways of antigen processing. Annu. Rev. Immunol 31, 443–473. doi: 10.1146/annurev-immunol-032712-095910 23298205 PMC4026165

[R9] Branchini BR, Ablamsky DM, Davis AL, Southworth TL, Butler B, Fan F, (2010). Red-emitting luciferases for bioluminescence reporter and imaging applications. Anal. Biochem 396, 290–297. doi: 10.1016/j.ab.2009.09.009 19748472

[R10] Bronte V, and Zanovello P (2005). Regulation of immune responses by L-arginine metabolism. Nat. Rev. Immunol 5, 641–654. doi: 10.1038/nri1668 16056256

[R11] Buitrago G, Pickering D, Ruscher R, Cobos Caceres C, Jones L, Cooper M, (2021). A netrin domain-containing protein secreted by the human hookworm Necator americanus protects against CD4 T cell transfer colitis. Transl. Res 232, 88–102. doi: 10.1016/j.trsl.2021.02.012 33676036

[R12] Camargo MM, Almeida IC, Pereira ME, Ferguson MA, Travassos LR, and Gazzinelli RT (1997). Glycosylphosphatidylinositol-anchored mucin-like glycoproteins isolated from Trypanosoma cruzi trypomastigotes initiate the synthesis of proinflammatory cytokines by macrophages. J. Immunol 158, 5890–5901. doi: 10.4049/jimmunol.158.12.5890 9190942

[R13] Cardoni RL, and Antunez MI (2004). Circulating levels of cyclooxygenase metabolites in experimental Trypanosoma cruzi infections. Mediat. Inflammation 13, 235–240. doi: 10.1080/09637480400003022 PMC1781569 15545053

[R14] Cerbán FM, Stempin CC, Volpini X, Silva EAC, Gea S, and Motran CC (2020). Signaling pathways that regulate Trypanosoma cruzi infection and immune response. Biochim. Biophys. Acta Mol. basis Dis 1866, 165707. doi: 10.1016/j.bbadis.2020.165707 32004621

[R15] Corral RS, Guerrero NA, Cuervo H, Gironès N, and Fresno M (2013). Trypanosoma cruzi infection and endothelin-1 cooperatively activate pathogenic inflammatory pathways in cardiomyocytes. PloS Negl. Trop. Dis 7, e2034. doi: 10.1371/journal.pntd.0002034 23409199 PMC3566987

[R16] Council, National Research. (2011). Guide for the care and use of laboratory animals 8th ed. (Washington, DC.: National Academy Press).

[R17] Croese J, Giacomin P, Navarro S, Clouston A, McCann L, Dougall A, (2015). Experimental hookworm infection and gluten microchallenge promote tolerance in celiac disease. J. Allergy Clin. Immunol 135, 508–516. doi: 10.1016/j.jaci.2014.07.022 25248819

[R18] Croese J, Wood MJ, Melrose W, and Speare R (2006). Allergy controls the population density of Necator americanus in the small intestine. Gastroenterology 131, 402–409. doi: 10.1053/j.gastro.2006.05.019 16890593

[R19] Cuellar C, Wu W, and Mendez S (2009). The hookworm tissue inhibitor of metalloproteases (Ac-TMP-1) modifies dendritic cell function and induces generation of CD4 and CD8 suppressor T cells. PloS Negl. Trop. Dis 3, e439. doi: 10.1371/journal.pntd.0000439 19468296 PMC2678263

[R20] Disease GBD, Injury I, and Prevalence C (2016). Global, regional, and national incidence, prevalence, and years lived with disability for 310 diseases and injuries, 1990-2015: a systematic analysis for the Global Burden of Disease Study 2015. Lancet 388, 1545–1602. doi: 10.1016/S0140-6736(16)31678-6 27733282 PMC5055577

[R21] Dos-Santos AL, Carvalho-Kelly LF, Dick CF, and Meyer-Fernandes JR (2016). Innate immunomodulation to trypanosomatid parasite infections. Exp. Parasitol 167, 67–75. doi: 10.1016/j.exppara.2016.05.005 27223816

[R22] Dubois RN, Abramson SB, Crofford L, Gupta RA, Simon LS, Van De Putte LB, (1998). Cyclooxygenase in biology and disease. FASEB J. Off. Publ. Fed. Am. Soc Exp. Biol 12 (12), 1063–1073. 9737710

[R23] Elliott DE, Summers RW, and Weinstock JV (2007). Helminths as governors of immune-mediated inflammation. Int. J. Parasitol 37 (5), 457–464. doi: 10.1016/j.ijpara.2006.12.009 17313951

[R24] Ferreira I, Pickering DA, Troy S, Croese J, Loukas A, and Navarro S (2017). Suppression of inflammation and tissue damage by a hookworm recombinant protein in experimental colitis. Clin. Transl. Immunol 6 (10), e157. doi: 10.1038/cti.2017.42 PMC5671989 29114386

[R25] Ferreira IB, Smyth D, Gaze S, Aziz A, Giacomin P, Ruyssers N, (2013). Hookworm excretory/secretory products induce interleukin-4 (IL-4)+ IL-10+ CD4+ T cell responses and suppress pathology in a mouse model of colitis. Infect. Immun 81 (6), 2104–2111. doi: 10.1128/IAI.00563-12 23545299 PMC3676036

[R26] Fresno M, and Gironès N (2021). Myeloid-derived suppressor cells in trypanosoma cruzi infection. Front. Cell. Infect. Microbiol 11, 737364. doi: 10.3389/fcimb.2021.737364 34513737 PMC8430253

[R27] GBD 2016 Disease and Injury Incidence and Prevalence Collaborators. (2017). Global, regional, and national incidence, prevalence, and years lived with disability for 328 diseases and injuries for 195 countries, 1990-2016: a systematic analysis for the Global Burden of Disease Study 2016. Lancet (London England) 390, 1211–1259. doi: 10.1016/S0140-6736(17)32154-2 28919117 PMC5605509

[R28] Gil-Jaramillo N, Motta FN, Favali CBF, Bastos IMD, and Santana JM (2016). Dendritic cells: A double-edged sword in immune responses during chagas disease. Front. Microbiol 7, 1076. doi: 10.3389/fmicb.2016.01076 27471496 PMC4943928

[R29] Ginhoux F, and Guilliams M (2016). Tissue-resident macrophage ontogeny and homeostasis. Immunity 44, 439–449. doi: 10.1016/j.immuni.2016.02.024 26982352

[R30] Guedes PM, Gutierrez FR, Silva GK, Dellalibera-Joviliano R, Rodrigues GJ, Bendhack LM, (2012). Deficient regulatory T cell activity and low frequency of IL-17-producing T cells correlate with the extent of cardiomyopathy in human Chagas’ disease. PloS Negl. Trop. Dis 6, e1630. doi: 10.1371/journal.pntd.0001630 22545173 PMC3335880

[R31] Guerrero NA, Camacho M, Vila L, Íñiguez MA, Chillón-Marinas C, Cuervo H, (2015). Cyclooxygenase-2 and Prostaglandin E2 Signaling through Prostaglandin Receptor EP-2 Favor the Development of Myocarditis during Acute Trypanosoma cruzi Infection. PloS Negl. Trop. Dis 9, e0004025. doi: 10.1371/journal.pntd.0004025 26305786 PMC4549243

[R32] He J, Li X, Zhuang J, Han J, Luo G, Yang F, (2018). Blocking matrix metalloproteinase-9 abrogates collagen-induced arthritis via inhibiting dendritic cell migration. J. Immunol 201, 3514–3523. doi: 10.4049/jimmunol.1800412 30397034

[R33] Higuchi ML, De Morais CF, Pereira Barreto AC, Lopes EA, Stolf N, and Bellotti G (1987). The role of active myocarditis in the development of heart failure in chronic Chagas’ disease: a study based on endomyocardial biopsies. Clin. Cardiol 10, 665–670. doi: 10.1002/clc.4960101113 3677499

[R34] Higuchi MD, Ries MM, Aiello VD, Benvenuti LA, Gutierrez PS, Bellotti G, (1997). Association of an increase in CD8+ T cells with the presence of Trypanosoma cruzi antigens in chronic, human, chagasic myocarditis. Am. J. Trop. Med. Hyg 56, 485–489. doi: 10.4269/ajtmh.1997.56.485 9180594

[R35] Higuchi Mde L, Gutierrez PS, Aiello VD, Palomino S, Bocchi E, Kalil J, (1993). Immunohistochemical characterization of infiltrating cells in human chronic chagasic myocarditis: comparison with myocardial rejection process. Virchows Arch. A Pathol. Anat Histopathol 423, 157–160. doi: 10.1007/BF01614765 7901937

[R36] Hoffman KA, Villar MJ, Poveda C, Bottazzi ME, Hotez PJ, Tweardy DJ, (2021). Signal transducer and activator of transcription-3 modulation of cardiac pathology in chronic chagasic cardiomyopathy. Front. Cell. Infect. Microbiol 11, 708325. doi: 10.3389/fcimb.2021.708325 34504808 PMC8421853

[R37] Hoffman KA, Reynolds C, Bottazzi ME, Hotez P, and Jones K (2019). Improved biomarker and imaging analysis for characterizing progressive cardiac fibrosis in a mouse model of chronic chagasic cardiomyopathy. J. Am. Heart Assoc 8, e013365. doi: 10.1161/JAHA.119.013365 31718442 PMC6915297

[R38] Hölscher C, Köhler G, Müller U, Mossmann H, Schaub GA, and Brombacher F (1998). Defective nitric oxide effector functions lead to extreme susceptibility of Trypanosoma cruzi-infected mice deficient in gamma interferon receptor or inducible nitric oxide synthase. Infect. Immun 66, 1208–1215. doi: 10.1128/IAI.66.3.1208-1215.1998 9488415 PMC108035

[R39] Huang B, Pan P-Y, Li Q, Sato AI, Levy DE, Bromberg J, (2006). Gr-1 +CD115+ immature myeloid suppressor cells mediate the development of tumor-induced T regulatory cells and T-cell anergy in tumor-bearing host. Cancer Res. 66, 1123–1131. doi: 10.1158/0008-5472.CAN-05-1299 16424049

[R40] Hübner MP, Shi Y, Torrero MN, Mueller E, Larson D, Soloviova K, (2012). Helminth protection against autoimmune diabetes in nonobese diabetic mice is independent of a type 2 immune shift and requires TGF-β. J. Immunol 188, 559–568. doi: 10.4049/jimmunol.1100335 22174447 PMC3253252

[R41] Jackson Y, Alirol E, Getaz L, Wolff H, Combescure C, and Chappuis F (2010). Tolerance and safety of nifurtimox in patients with chronic chagas disease. Clin. Infect. Dis. an Off. Publ. Infect. Dis. Soc Am 51, e69–e75. doi: 10.1086/656917 20932171

[R42] Keating SM, Deng X, Fernandes F, Cunha-Neto E, Ribeiro AL, Adesina B, (2015). Inflammatory and cardiac biomarkers are differentially expressed in clinical stages of Chagas disease. Int. J. Cardiol 199, 451–459. doi: 10.1016/j.ijcard.2015.07.040 26277551 PMC4868386

[R43] Konduri V, Li D, Halpert MM, Liang D, Liang Z, Chen Y, (2016). Chemo-immunotherapy mediates durable cure of orthotopic Kras(G12D)/p53(−/−) pancreatic ductal adenocarcinoma. Oncoimmunology 5, e1213933. doi: 10.1080/2162402X.2016.1213933 27757308 PMC5048769

[R44] Konduri V, Halpert MM, Liang D, Levitt JM, Cruz-Chan JV, Zhan B, (2017). Genetic adjuvantation of a cell-based therapeutic vaccine for amelioration of chagasic cardiomyopathy. Infect. Immun 85(9), e00127–17. doi: 10.1128/IAI.00127-17 28674032 PMC5563592

[R45] Langdon K, Buitrago G, Pickering D, Giacomin P, Loukas A, and Haleagrahara N (2022). Na-AIP-1 secreted by human hookworms suppresses collagen-induced arthritis. Inflammopharmacology 30, 527–535. doi: 10.1007/s10787-021-00909-5 35031905

[R46] Laucella SA, Postan M, Martin D, Hubby Fralish B, Albareda MC, Alvarez MG, (2004). Frequency of interferon- gamma -producing T cells specific for Trypanosoma cruzi inversely correlates with disease severity in chronic human Chagas disease. J. Infect. Dis 189, 909–918. doi: 10.1086/381682 14976609

[R47] Lee M, Rey K, Besler K, Wang C, and Choy J (2017). Immunobiology of nitric oxide and regulation of inducible nitric oxide synthase. Results Probl. Cell Differ 62, 181–207. doi: 10.1007/978-3-319-54090-0_8 28455710

[R48] Lewis MD, Francisco AF, Taylor MC, and Kelly JM (2015). A new experimental model for assessing drug efficacy against Trypanosoma cruzi infection based on highly sensitive in *vivo* imaging. J. Biomol Screen 20, 36–43. doi: 10.1177/1087057114552623 25296657 PMC4361455

[R49] Li H, Zhang G-X, Chen Y, Xu H, Fitzgerald DC, Zhao Z, (2008). CD11c +CD11b+ dendritic cells play an important role in intravenous tolerance and the suppression of experimental autoimmune encephalomyelitis. J. Immunol 181, 2483–2493. doi: 10.4049/jimmunol.181.4.2483 18684939 PMC2676731

[R50] Li H, Wang S, Zhan B, He W, Chu L, Qiu D, (2017). Therapeutic effect of Schistosoma japonicum cystatin on bacterial sepsis in mice. Parasitol. Vectors 10, 222. doi: 10.1186/s13071-017-2162-0 PMC5422996 28482922

[R51] Liang D, Tian L, You R, Halpert MM, Konduri V, Baig YC, (2017). AIMp1 potentiates T(H)1 polarization and is critical for effective antitumor and antiviral immunity. Front. Immunol 8, 1801. doi: 10.3389/fimmu.2017.01801 29379495 PMC5775236

[R52] López L, Arai K, Giménez E, Jiménez M, Pascuzo C, Rodríguez-Bonfante C, (2006). [C-reactive protein and interleukin-6 serum levels increase as Chagas disease progresses towards cardiac failure]. Rev. Esp. Cardiol 59 (1), 50–56. doi: 10.1016/S1885-5857(06)60048-0 16434004

[R53] MaChado FS, Mukherjee S, Weiss LM, Tanowitz HB, and Ashton AW (2011). Bioactive lipids in Trypanosoma cruzi infection. Adv. Parasitol 76, 1–31. doi: 10.1016/B978-0-12-385895-5.00001-3 21884885 PMC3564251

[R54] Magalhães LM, Villani FN, Nunes Mdo C, Gollob KJ, Rocha MO, and Dutra WO. (2013). High interleukin 17 expression is correlated with better cardiac function in human Chagas disease. J. Infect. Dis 207, 661–665. doi: 10.1093/infdis/jis724 23204182 PMC3611763

[R55] Malvezi AD, Panis C, da Silva RV, de Freitas RC, Lovo-Martins MI, Tatakihara VL, (2014a). Inhibition of cyclooxygenase-1 and cyclooxygenase-2 impairs Trypanosoma cruzi entry into cardiac cells and promotes differential modulation of the inflammatory response. Antimicrob. Agents Chemother. 58, 6157–6164. doi: 10.1128/AAC.02752-14 25092706 PMC4187892

[R56] Malvezi AD, da Silva RV, Panis C, Yamauchi LM, Lovo-Martins MI, Zanluqui NG, (2014b). Aspirin modulates innate inflammatory response and inhibits the entry of Trypanosoma cruzi in mouse peritoneal macrophages. Mediat. Inflammation 2014, 580919. doi: 10.1155/2014/580919 PMC4089847 25045211

[R57] Mantovani A, Biswas SK, Galdiero MR, Sica A, and Locati M (2013). Macrophage plasticity and polarization in tissue repair and remodelling. J. Pathol 229, 176–185. doi: 10.1002/path.4133 23096265

[R58] Mariano FS, Gutierrez FRS, Pavanelli WR, Milanezi CM, Cavassani KA, Moreira AP, (2008). The involvement of CD4+CD25+ T cells in the acute phase of Trypanosoma cruzi infection. MicrobesInfect. 10, 825–833. doi: 10.1016/j.micinf.2008.04.009 18538611

[R59] McSorley HJ, Hewitson JP, and Maizels RM (2013). Immunomodulation by helminth parasites: defining mechanisms and mediators. Int. J. Parasitol 43, 301–310. doi: 10.1016/j.ijpara.2012.11.011 23291463

[R60] Molina I, Salvador F, Sanchez-Montalva A, Trevino B, Serre N, Sao Aviles A, (2015). Toxic profile of benznidazole in patients with chronic chagas disease: risk factors and comparison of the product from two different manufacturers. Antimicrob. Agents Chemother 59, 6125–6131. doi: 10.1128/AAC.04660-14 26195525 PMC4576116

[R61] Molina-Berríos A, Campos-Estrada C, Henriquez N, Faúndez M, Torres G, Castillo C, (2013). Protective role of acetylsalicylic acid in experimental Trypanosoma cruzi infection: evidence of a 15-epi-lipoxin A_4_-mediated effect. PloS Negl. Trop. Dis 7, e2173. doi: 10.1371/journal.pntd.0002173 23638194 PMC3630130

[R62] Morillo CA, Marin-Neto JA, Avezum A, Sosa-Estani S, Rassi A Jr, Rosas F, (2015). Randomized trial of benznidazole for chronic chagas’ Cardiomyopathy. N. Engl. J. Med 373, 1295–1306. doi: 10.1056/NEJMoa1507574 26323937

[R63] Mukherjee S, Machado FS, Huang H, Oz HS, Jelicks LA, Prado CM, (2011). Aspirin treatment of mice infected with Trypanosoma cruzi and implications for the pathogenesis of Chagas disease. PloS One 6, e16959. doi: 10.1371/journal.pone.0016959 21347238 PMC3039660

[R64] Muñoz-Fernández MA, Fernández MA, and Fresno M (1992). Synergism between tumor necrosis factor-alpha and interferon-gamma on macrophage activation for the killing of intracellular Trypanosoma cruzi through a nitric oxide-dependent mechanism. Eur. J. Immunol 22, 301–307. doi: 10.1002/eji.1830220203 1537373

[R65] Nair MG, and Herbert DR (2016). Immune polarization by hookworms: taking cues from T helper type 2, type 2 innate lymphoid cells and alternatively activated macrophages. Immunology 148, 115–124. doi: 10.1111/imm.12601 26928141 PMC4863575

[R66] Navarro S, Pickering DA, Ferreira IB, Jones L, Ryan S, Troy S, (2016). Hookworm recombinant protein promotes regulatory T cell responses that suppress experimental asthma. Sci. Transl. Med 8 (362), 362ra143. doi: 10.1126/scitranslmed.aaf8807 27797959

[R67] Piron M, Fisa R, Casamitjana N, Lopez-Chejade P, Puig L, and Verges M (2007). Development of a real-time PCR assay for Trypanosoma cruzi detection in blood samples. Acta Trop. 103 (3), 195–200. doi: 10.1016/j.actatropica.2007.05.019 17662227

[R68] Reis MM, Mde Higuchi L, Benvenuti LA, Aiello VD, Gutierrez PS, Bellotti G, (1997). An in *situ* quantitative immunohistochemical study of cytokines and IL-2R+ in chronic human chagasic myocarditis: correlation with the presence of myocardial Trypanosoma cruzi antigens. Clin. Immunol. Immunopathol 83 (2), 165–172. doi: 10.1006/clin.1997.4335 9143377

[R69] Rodrigues JPF, Caldas IS, Gonçalves RV, Almeida LA, Souza RLM, and Novaes RD. (2017). S. mansoni-T. cruzi co-infection modulates arginase-1/iNOS expression, liver and heart disease in mice. Nitric. Oxide 66, 43–52. doi: 10.1016/j.niox.2017.02.013 28268114

[R70] Rossi MA, Ramos SG, and Bestetti RB (2003). Chagas’ heart disease: clinical-pathological correlation. Front. Biosci 8, e94–109. doi: 10.2741/948 12456334

[R71] Ruiz-Sanchez R, Leon MP, Matta V, Reyes PA, Lopez R, Jay D, (2005). Trypanosoma cruzi isolates from Mexican and Guatemalan acute and chronic chagasic cardiopathy patients belong to Trypanosoma cruzi I. Mem Inst Oswaldo Cruz 100 (3), 281–283. doi: 10.1590/S0074-02762005000300012 16113869

[R72] Ruyssers NE, De Winter BY, De Man JG, Loukas A, Pearson MS, Weinstock JV, (2009). Therapeutic potential of helminth soluble proteins in TNBS-induced colitis in mice. Inflammation Bowel Dis. 15 (4), 491–500. doi: 10.1002/ibd.20787 19023900

[R73] Sabino EC, Ribeiro AL, Salemi VMC, Oliveira C, Antunes AP, Menezes MM, (2013). Ten-Year incidence of chagas cardiomyopathy among asymptomatic trypanosoma cruzi-seropositive former blood donors. Circulation 127 (10), 1105–1115. doi: 10.1161/CIRCULATIONAHA.112.123612 23393012 PMC3643805

[R74] Santos E, de S, de Aragão-França LS, Meira CS, Cerqueira JV, Vasconcelos JF, Nonaka CKV, (2020). Tolerogenic dendritic cells reduce cardiac inflammation and fibrosis in chronic chagas disease. Front. Immunol 11, 488. doi: 10.3389/fimmu.2020.00488 32318058 PMC7154094

[R75] Shapouri-Moghaddam A, Mohammadian S, Vazini H, Taghadosi M, Esmaeili S-A, Mardani F, (2018). Macrophage plasticity, polarization, and function in health and disease. J. Cell. Physiol 233 (9), 6425–6440. doi: 10.1002/jcp.26429 PMC13482560 29319160

[R76] Sheel M, and Engwerda CR (2012). The diverse roles of monocytes in inflammation caused by protozoan parasitic diseases. Trends Parasitol. 28, 408–416. doi: 10.1016/j.pt.2012.07.008 22951424

[R77] Silva JS, Morrissey PJ, Grabstein KH, Mohler KM, Anderson D, and Reed SG. (1992). Interleukin 10 and interferon gamma regulation of experimental Trypanosoma cruzi infection. J. Exp. Med 175 (1), 169–174. doi: 10.1084/jem.175.1.169 1730915 PMC2119081

[R78] Sousa GR, Gomes JA, Damasio MP, Nunes MC, Costa HS, Medeiros NI, (2017). The role of interleukin 17-mediated immune response in Chagas disease: High level is correlated with better left ventricular function. PloS One 12 (3), e0172833. doi: 10.1371/journal.pone.0172833 28278264 PMC5344340

[R79] Sousa GR, Gomes JA, Fares RC, Damasio MP, Chaves AT, Ferreira KS, (2014). Plasma cytokine expression is associated with cardiac morbidity in chagas disease. PloS One 9 (3), e87082. doi: 10.1371/journal.pone.0087082 24603474 PMC3945957

[R80] Tarleton RL (1990). Depletion of CD8+ T cells increases susceptibility and reverses vaccine-induced immunity in mice infected with Trypanosoma cruzi. J. Immunol 144, 717–724. doi: 10.4049/jimmunol.144.2.717 2104903

[R81] Tarleton RL (2007). Immune system recognition of Trypanosoma cruzi. Curr. Opin. Immunol 19, 430–434. doi: 10.1016/j.coi.2007.06.003 17651955

[R82] Thwe PM, and Amiel E (2018). The role of nitric oxide in metabolic regulation of Dendritic cell immune function. Cancer Lett. 412, 236–242. doi: 10.1016/j.canlet.2017.10.032 29107106 PMC5699934

[R83] Tzelepis F, de Alencar BCG, Penido MLO, Gazzinelli RT, Persechini PM, and Rodrigues MM (2006). Distinct kinetics of effector CD8+ cytotoxic T cells after infection with Trypanosoma cruzi in naive or vaccinated mice. Infect. Immun 74 (4), 2477–2481. doi: 10.1128/IAI.74.4.2477-2481.2006 16552083 PMC1418894

[R84] Urbina JA (2010). Specific chemotherapy of Chagas disease: relevance, current limitations and new approaches. Acta Trop. 115, 55–68. doi: 10.1016/j.actatropica.2009.10.023 19900395

[R85] van Riet E, Hartgers FC, and Yazdanbakhsh M (2007). Chronic helminth infections induce immunomodulation: consequences and mechanisms. Immunobiology 212, 475–490. doi: 10.1016/j.imbio.2007.03.009 17544832

[R86] Vespa GN, Cunha FQ, and Silva JS (1994). Nitric oxide is involved in control of Trypanosoma cruzi-induced parasitemia and directly kills the parasite. vitro. Infect. Immun 62, 5177–5182. doi: 10.1128/iai.62.11.5177-5182.1994 7523307 PMC303244

[R87] Villanueva-Lizama LE, Cruz-Chan JV, Versteeg L, Teh-Poot CF, Hoffman K, Kendricks A, (2020). TLR4 agonist protects against Trypanosoma cruzi acute lethal infection by decreasing cardiac parasite burdens. Parasite Immunol. 42 (10), e12769. doi: 10.1111/pim.12769 32592180

[R88] Viotti R, Vigliano C, Lococo B, Alvarez MG, Petti M, and Bertocchi G (2009). Side effects of benznidazole as treatment in chronic Chagas disease: fears and realities. Expert Rev. Anti Infect. Ther 7 (2), 157–163. doi: 10.1586/14787210.7.2.157 19254164

[R89] Wan X, Wen JJ, Koo SJ, Liang LY, and Garg NJ (2016). SIRT1-PGC1alpha-NFkappaB Pathway of Oxidative and Inflammatory Stress during Trypanosoma cruzi Infection: Benefits of SIRT1-Targeted Therapy in Improving Heart Function in Chagas Disease. PloS Pathog. 12, e1005954. doi: 10.1371/journal.ppat.1005954 27764247 PMC5072651

[R90] Willingham SB, Volkmer J-P, Gentles AJ, Sahoo D, Dalerba P, Mitra SS, (2012). The CD47-signal regulatory protein alpha (SIRPa) interaction is a therapeutic target for human solid tumors. Proc. Natl. Acad. Sci. U. S. A 109 (107), 6662–6667. doi: 10.1073/pnas.1121623109 22451913 PMC3340046

[R91] Wirth JJ, Kierszenbaum F, Sonnenfeld G, and Zlotnik A (1985). Enhancing effects of gamma interferon on phagocytic cell association with and killing of Trypanosoma cruzi. Infect. Immun 49, 61–66. doi: 10.1128/iai.49.1.61-66.1985 3924832 PMC262058

[R92] Wu Z-H, Li N, Mei X-F, Chen J, Wang X-Z, and Guo T-T (2022). Preclinical characterization of the novel anti-SIRPa antibody BR105 that targets the myeloid immune checkpoint. J. Immunother. Cancer 10 (3), e004054. doi: 10.1136/jitc-2021-004054 35256517 PMC8905892

[R93] Zhan B, Badamchian M, Meihua B, Ashcom J, Feng J, Hawdon J, (2002). Molecular cloning and purification of Ac-TMP, a developmentally regulated putative tissue inhibitor of metalloprotease released in relative abundance by adult Ancylostoma hookworms. Am. J. Trop. Med. Hyg 66, 238–244. doi: 10.4269/ajtmh.2002.66.238 12139214

[R94] Zhan B, Gupta R, Wong SP, Bier S, Jiang D, and Goud G (2008). Molecular cloning and characterization of Ac-TMP-2, a tissue inhibitor of metalloproteinase secreted by adult Ancylostoma caninum. Mol. Biochem. Parasitol 162 (2), 142–148. doi: 10.1016/j.molbiopara.2008.08.008 18804124

