# Supplementary material for: Immunomodulatory proteins from hookworms reduce cardiac inflammation and modulate regulatory responses in a mouse model of chronic Trypanosoma cruzi infection
Source: Front Parasitol. Author manuscript; Available in PMC 2024 Jan 18. (PMC10795693; doi:10.3389/fpara.2023.1244604)
Supplement: Supplementary material [file NIHMS1957857-supplement-Supplementary_material.docx]

**Supplemental Data**

**Immunomodulatory proteins from hookworms reduce cardiac inflammation and modulate regulatory responses in a mouse model of chronic Trypanosoma cruzi infection**

Kathryn M. Jones^1,2$,^ Bin Zhan^1,2^, Keenan J Ernste^3,^ Maria Jose Villar^1,2^, Nalini Bisht^3^, Duc Nguyen^4^, Li-Yen Chang^5^, Cristina Poveda^1,2^, Gonteria J Robinson^6^, Akshar J Trivedi^3^, Colby J Hofferek^3^, William K. Decker^3,7,9^, Vanaja Konduri^3,7,8^ ^$^

^1^National School of Tropical Medicine, Department of Pediatrics, Baylor College of Medicine, Houston, TX, United States

^2^Texas Children’s Hospital Center for Vaccine Development, Houston, TX, United States

^3^ Department of Pathology & Immunology, Baylor College of Medicine, Houston, TX, United States

^4^ Center for Comparative Medicine, Baylor College of Medicine, Houston, TX, United States

^5^ Department of Medical Microbiology, Universiti Malaya, Kuala Lumpur, Malaysia

^6^ Molecular & Human Genetics Department, Baylor College of Medicine, Houston, TX, United States

^7^ Dan L Duncan Cancer, Baylor College of Medicine, Houston, TX, United States

^8^ Center for Cell and Gene Therapy, Baylor College of Medicine, Houston, TX, United States

^$^ Corresponding Authors [kathrynj@bcm.edu](mailto:kathrynj@bcm.edu); [konduri@bcm.edu](mailto:konduri@bcm.edu)

**Keywords:** Hookworm, anti-inflammatory, Trypanosoma cruzi, myocarditis, immunomodulatory


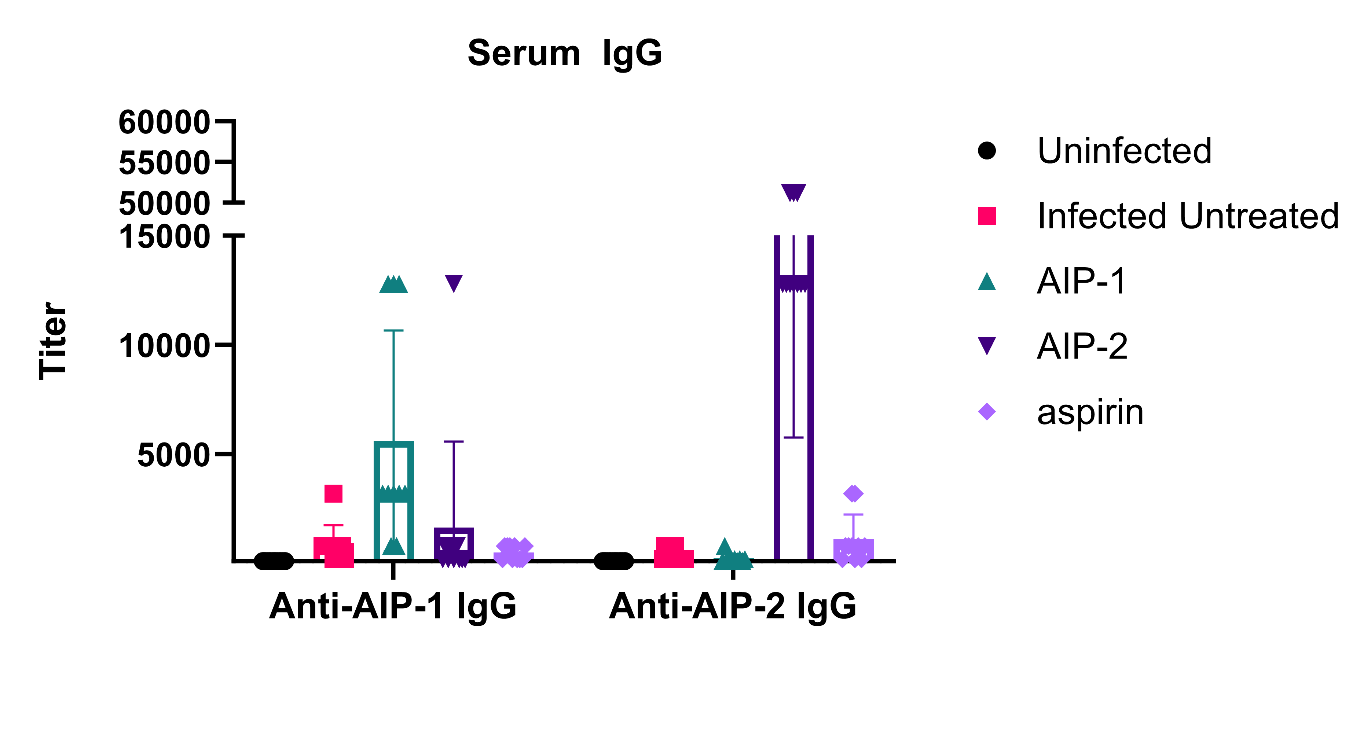


**Supplemental Figure 1**: Serum IgG titers. Anti-AIP-1 (left) and anti-AIP-2 (right) total IgG titers were measured from the serum of mice at study endpoint by indirect ELISA.


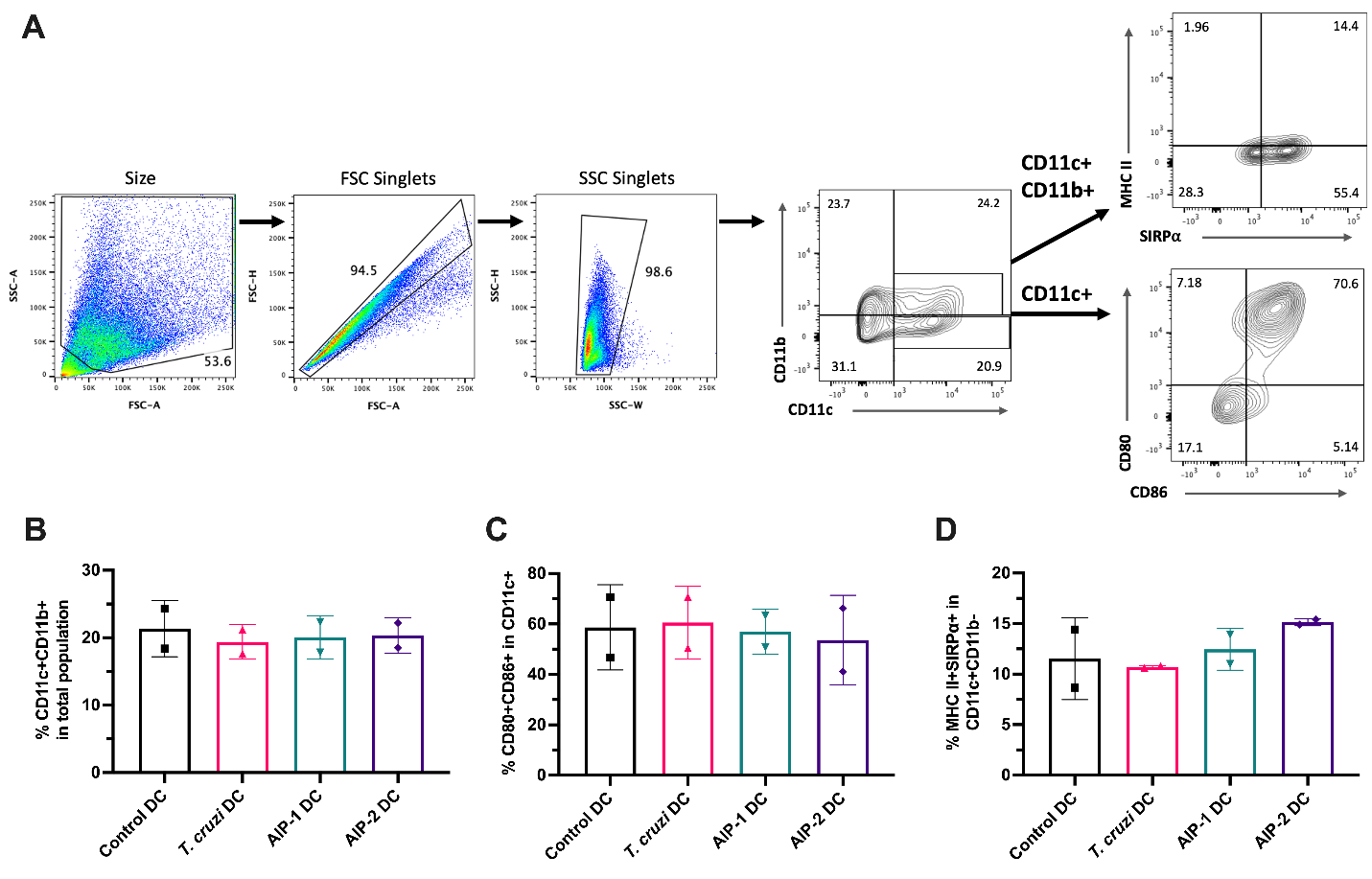


**Supplemental Figure 2:** Bone marrow derived DC phenotyping prior to DC-splenocyte co-cultures. Healthy wild type mouse BMDC were incubated with 10µg/mL *T.* cruzi parasite lysate, 50μg/mL of AIP-1 protein, 50μg/mL of AIP-2 protein, or no protein (control). After three hours of antigenic incubation in serum starved media, the cells were replenished with complete media supplemented with pro-inflammatory maturation cocktail (GM-CSF, IL-4, TNFα, IL-1β , IL-6 and PGE2). 48 hours post maturation, DC were harvested and phenotyped by flow cytometry. Representative plots for the overall gating strategy is shown in (A).(B) shows the frequency of CD11c+CD11b+ cells within the total population for each DC treatment group. (C) shows the frequency of CD80+CD86+ cells within the CD11c+ population for each DC treatment group. (D) shows the frequency of MHC-II+SIRP**⍺** cells within the CD11c+CD11b+ populations of DC treatment groups. Error bars are defined by mean with SD.


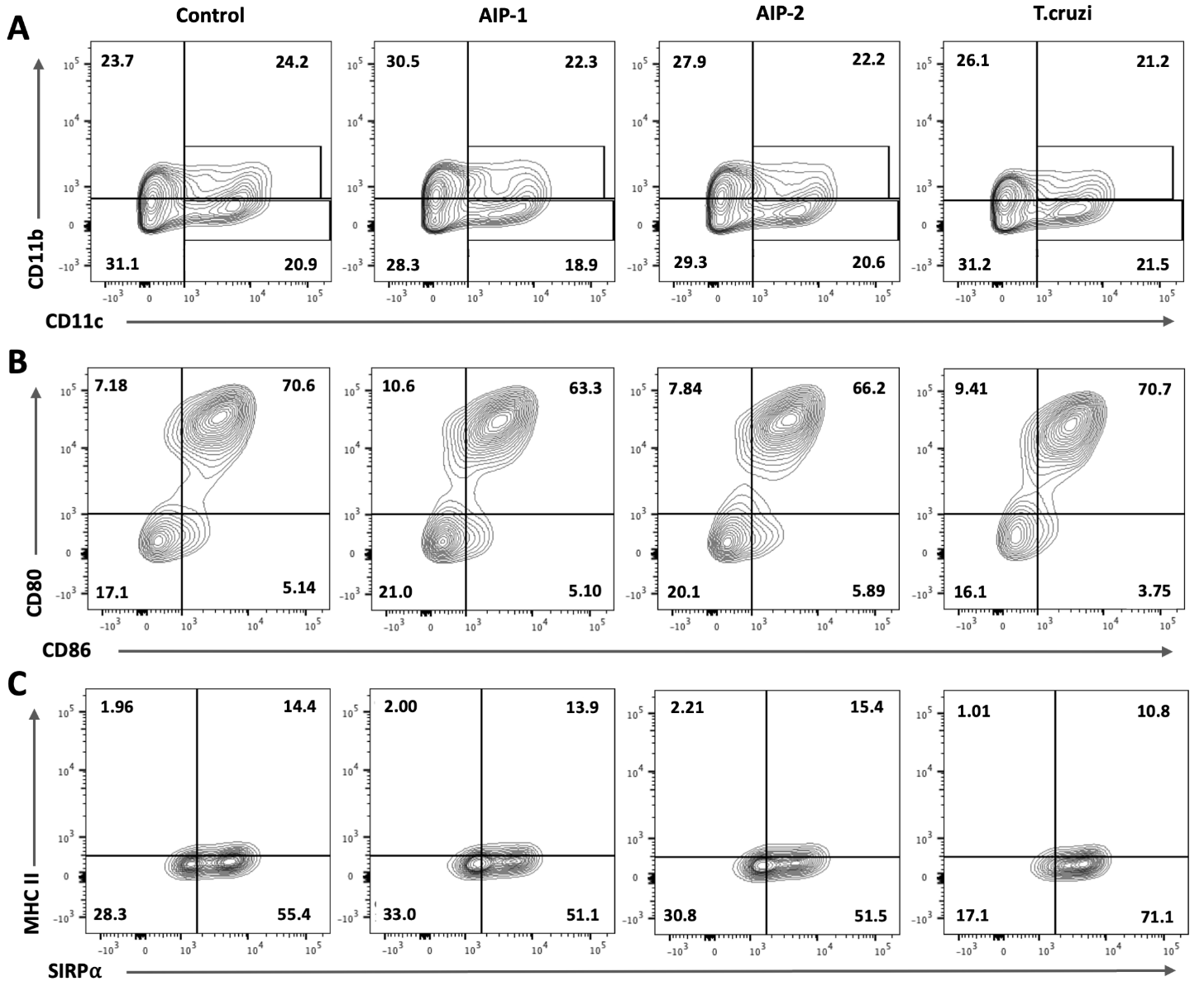


**Supplemental Figure 3**: Representative flow cytometry plots for DC phenotyping prior to DC-splenocyte co-culture. Mouse bone marrow-derived DC were incubated with 10μg/mL of *T.cruzi* parasite lysate, 50μg/mL of AIP-1, or 50μg/mL of AIP-2 hookworm proteins. After three hours of antigenic incubation in serum starved media, the cells were replenished with complete media supplemented with maturation cocktail (GM-CSF, IL-4, TNFα, IL-1β, IL-6 and PGE2). 48 hours post maturation, DC were harvested and analyzed by flow cytometry for (A) CD11c and CD11b expression within the total singlet population, (B) CD80 and CD86 expression within the CD11c+ population, and (C) MHC-II and SIRP⍺ expression within the CD11c+CD11b+ cell population (C). Comparisons were made with control DC that received pro-inflammatory cytokines but were not loaded with either *T.cruzi* parasite lysate, AIP-1 protein, or AIP-2 protein.


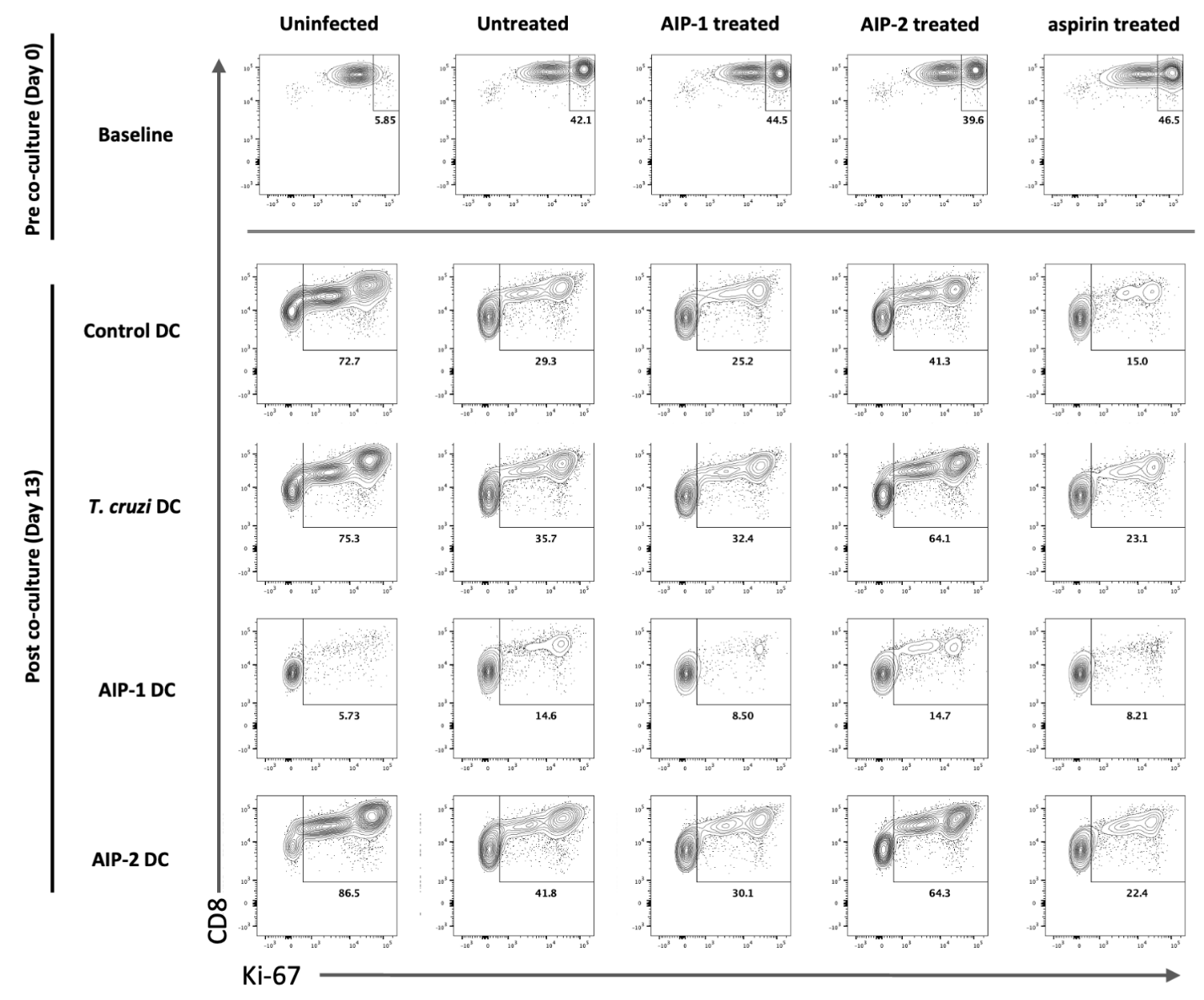


**Supplemental Figure 4**: Representative flow cytometry plots for Ki-67 expression analysis within the CD8+ T cell population of mouse splenocytes before and after co-culture with mouse DCs. Splenocytes harvested from uninfected-untreated, infected-untreated, infected-AIP-1 treated, infected-AIP-2 treated, or infected aspirin treated mice were co-cultured with murine DC loaded with AIP-1 protein, AIP-2 protein, *T. cruzi* lysate, or no protein (control DC). Flow cytometry analysis was performed on freshly isolated, unstimulated splenocytes (baseline) and again on the same population after 13 days of co-culture with DCs. Each gated population shown in the representative plots represents Ki-67+ cells within the CD8+ T cell population (CD3+CD8+).


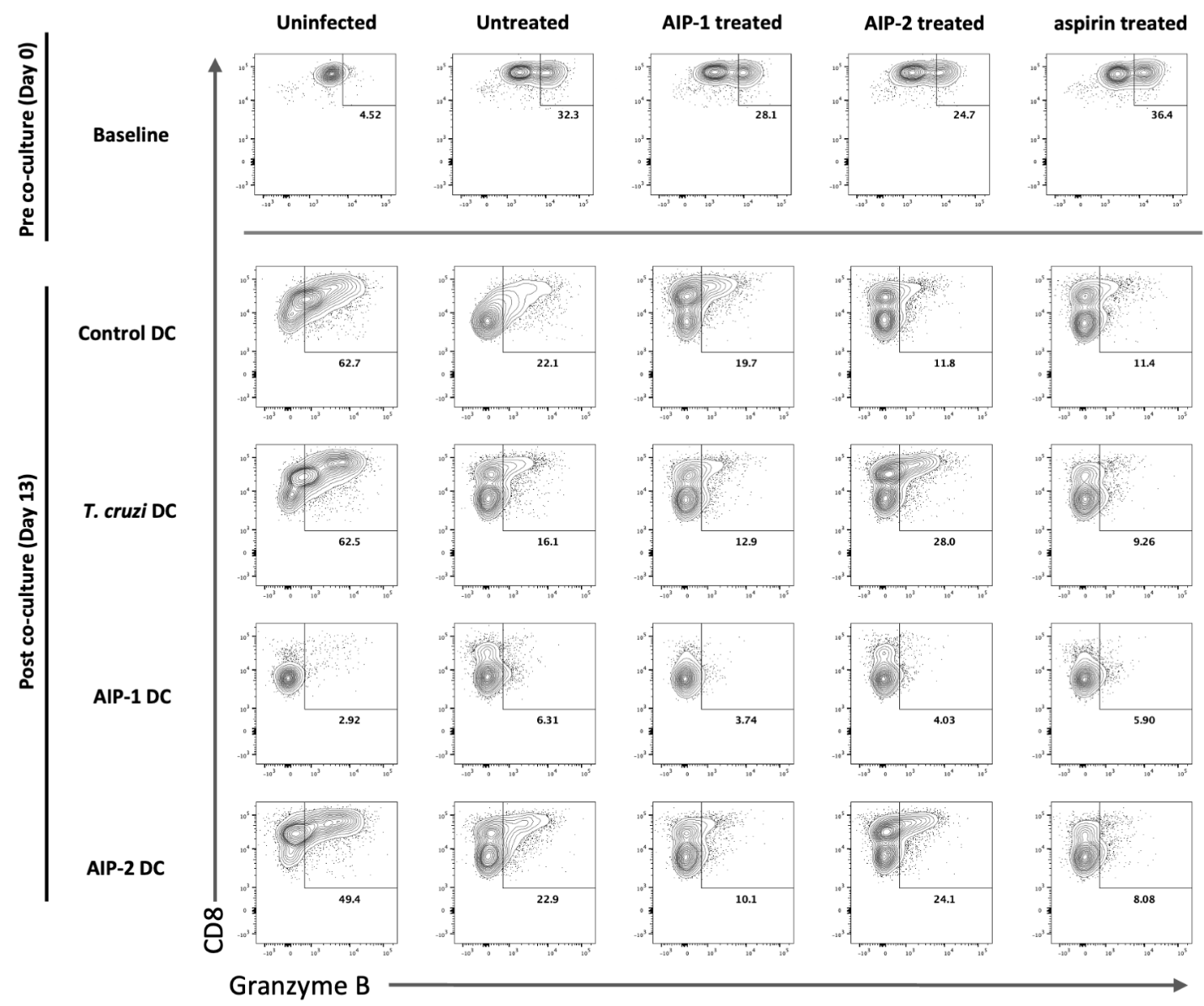


**Supplemental Figure 5**: Representative flow cytometry plots for Granzyme B expression analysis within the CD8+ T cell population of mouse splenocytes before and after co-culture with mouse DCs. Splenocytes harvested from uninfected-untreated, infected-untreated, infected-AIP-1 treated, infected-AIP-2 treated, or infected aspirin treated mice were co-cultured with murine DC loaded with AIP-1 protein, AIP-2 protein, *T. cruzi* lysate, or no protein (control DC). Flow cytometry analysis was performed on freshly isolated, unstimulated splenocytes (baseline) and again on the same population after 13 days of co-culture with DCs. Each gated population shown in the representative plots represents Granzyme B+ cells within the CD8+ T cell population (CD3+CD8+).


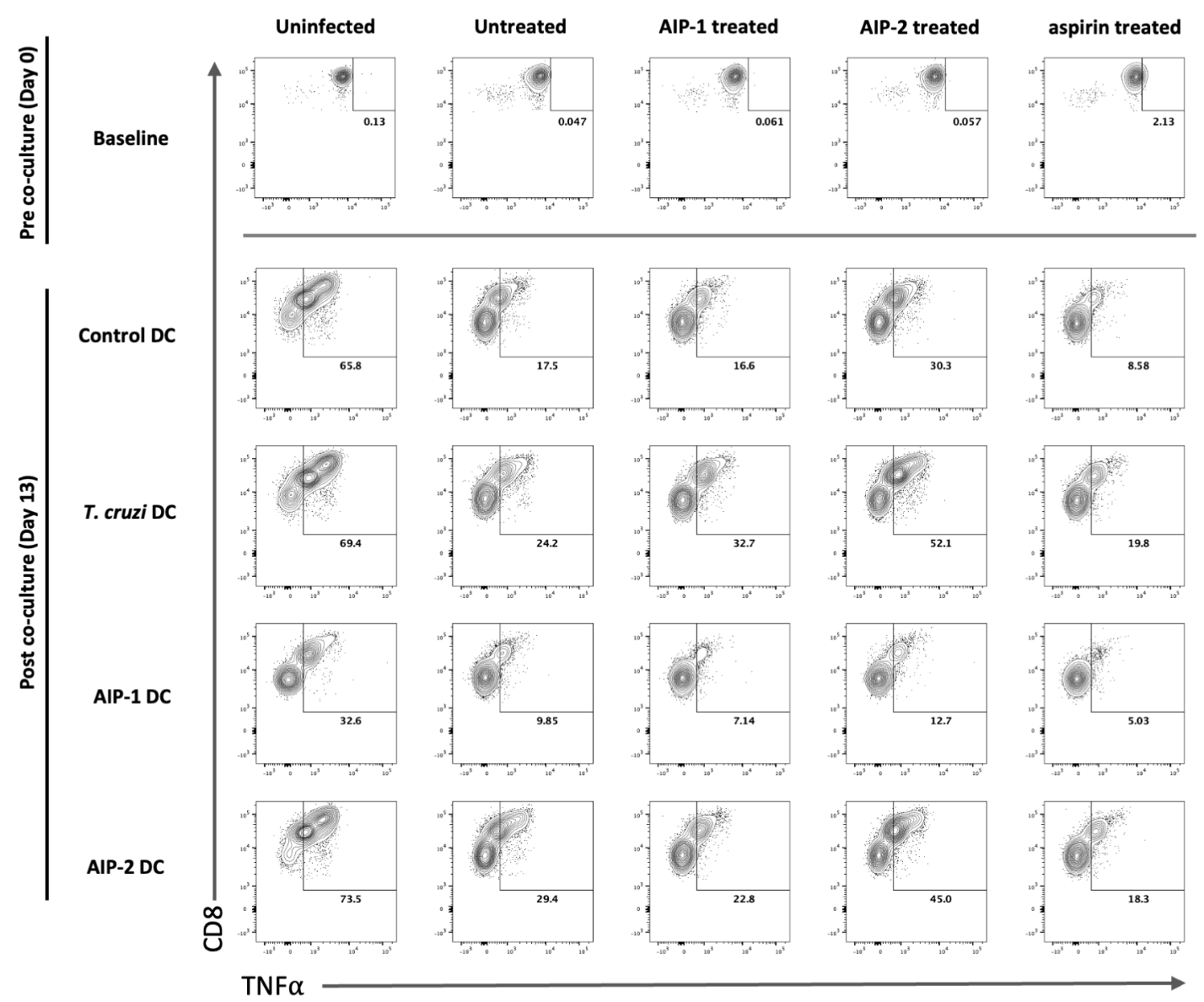


**Supplemental Figure 6**: Representative flow cytometry plots for TNFα expression analysis within the CD8+ T cell population of mouse splenocytes before and after co-culture with mouse DCs. Splenocytes harvested from uninfected-untreated, infected-untreated, infected-AIP-1 treated, infected-AIP-2 treated, or infected aspirin treated mice were co-cultured with murine DC loaded with AIP-1 protein, AIP-2 protein, *T. cruzi* lysate, or no protein (control DC). Flow cytometry analysis was performed on freshly isolated, unstimulated splenocytes (baseline) and again on the same population after 13 days of co-culture with DCs. Each gated population shown in the representative plots represents TNFα+ cells within the CD8+ T cell population (CD3+CD8+).


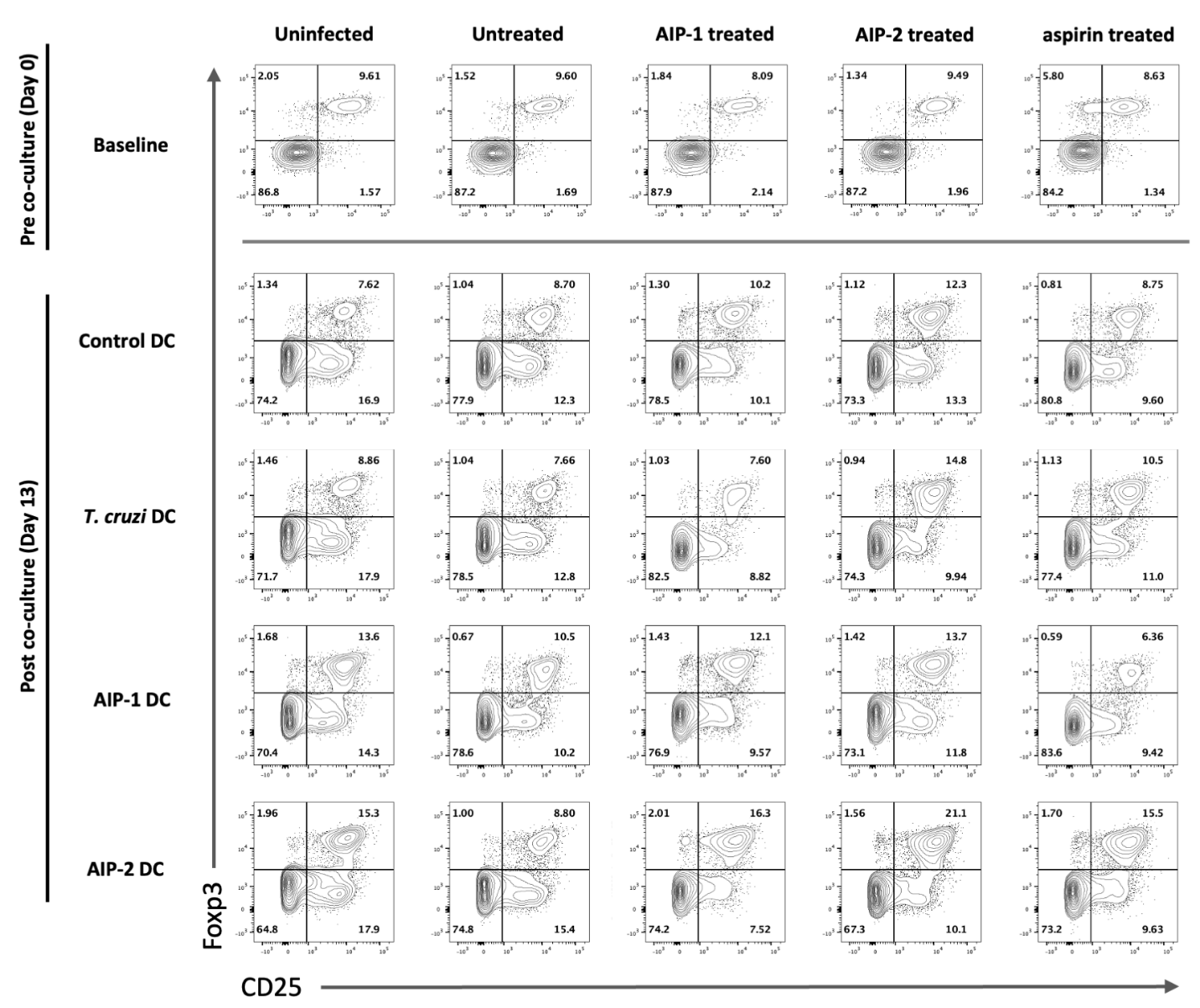


**Supplemental Figure 7**: Representative flow cytometry plots for Treg expression analysis within the CD4+ T cell population of mouse splenocytes before and after co-culture with mouse DCs. Splenocytes harvested from uninfected-untreated, infected-untreated, infected-AIP-1 treated, infected-AIP-2 treated, or infected aspirin treated mice were co-cultured with murine DC loaded with AIP-1 protein, AIP-2 protein, *T. cruzi* lysate, or no protein (control DC). Flow cytometry analysis was performed on freshly isolated, unstimulated splenocytes (baseline) and again on the same population after 13 days of co-culture with DCs. CD25+Foxp3+ cells within the CD4+ T cell population (CD3+CD4+) were considered Tregs.

**Supplemental Table 1**: Primer and Probe sequences for quantitative PCR.

| **Primer/ Probe** | **Sequence** |
| --- | --- |
| **Cruzi 1 (Forward Primer)** | 5’ ASTCGGCTGATCGTTTTCGA 3’ |
| **Cruzi 2 (Reverse Primer)** | 5’ AATTCCTCCAAGCAGCGGATA 3’ |
| **Cruzi 3 (Probe)** | 5’ 6-FAM CACACACTGGACACCAA MGB 3’ |
| **GAPDH Forward Primer** | 5’ CAA TGT GTC CGT CGT GGA TCT 3’ |
| **GAPDH Reverse Primer** | 5’ GTC CTC AGT GTA GCC CAA GAT G 3’ |
| **GAPDH Probe** | 5’ 6-FAM CGT GCC GCC TGG AGA AAC CTG CC MGB 3’ |

**Supplemental Table 2:** Gene reference numbers for RT-PCR

| **Gene** | **Reference Number** |
| --- | --- |
| **Arg1** | Mm00475988_m1 |
| **Mmp9** | Mm00442991_m1 |
| **Nos2** | Mm00440502_m1 |
| **Cox2** | Mm00478374_m1 |
| **STAT-1** | Mm00439518_m1 |
| **NFκ-β** | Mm00476361_m1 |

**Supplemental Table 3:** Antibodies and fluorochromes used for immunophenotyping of DCs and T cells.

| **Marker** | **Fluorophore** | **Clone** | **Catalog Number** | **Manufacturer** |
| --- | --- | --- | --- | --- |
| CD11c | PE/Cy5.5 | N418 | 117316 | Biolegend |
| CD11b​ | BV510​ | M1/70​ | 101245​ | Biolegend |
| CD8a​ | APC​ | 53-6.7​ | 100752​ | Biolegend |
| CD8a​ | BV510​ | 53-6.7​ | 100752​ | Biolegend |
| I-Ab​ (MHC II) | FITC​ | KH74​ | 115305 | Biolegend |
| CD172α (SIRPα)​ | PE​ | P84​ | 144011​ | Biolegend |
| CD80​ | PE​ | 16-10A1​ | 104707​ | Biolegend |
| CD86​ | BV650​ | GL-1​ | 105035​ | Biolegend |
| CD3 | PerCP/Cy5.5 | HIT3a | 300327 | Biolegend |
| CD4​ | FITC​ | RM5-4​ | 100510​ | Biolegend |
| Foxp3​ | PE​ | MF-14​ | 126404​ | Biolegend |
| Ki-67​ | APC​ | 16A8​ | 652406​ | Biolegend |
